# NR2F2 alleviates pulmonary fibrosis by inhibition of epithelial cell senescence

**DOI:** 10.1186/s12931-024-02777-3

**Published:** 2024-04-02

**Authors:** Ruyan Wan, Siqi Long, Shuaichen Ma, Peishuo Yan, Zhongzheng Li, Kai Xu, Hui Lian, Wenwen Li, Yudi Duan, Miaomiao Zhu, Lan Wang, Guoying Yu

**Affiliations:** 1 State Key Laboratory Cell Differentiation and Regulation, Henan International Joint Laboratory of Pulmonary Fibrosis, Henan center for outstanding overseas scientists of pulmonary fibrosis, College of Life Science, Institute of Biomedical Science, Pingyuan Laboratory, Henan Normal University, Xinxiang, 453007 Henan China; 2 College of Life Science, Henan Normal University, Xinxiang, Henan China

**Keywords:** NR2F2, Cell senescence, DNA damage, Epithelial-mesenchymal crosstalk, Pulmonary fibrosis

## Abstract

Idiopathic pulmonary fibrosis (IPF) is a chronic, progressive, fatal, and aging-associated interstitial lung disease with a poor prognosis and limited treatment options, while the pathogenesis remains elusive. In this study, we found that the expression of nuclear receptor subfamily 2 group F member 2 (NR2F2), a member of the steroid thyroid hormone superfamily of nuclear receptors, was reduced in both IPF and bleomycin-induced fibrotic lungs, markedly in bleomycin-induced senescent epithelial cells. Inhibition of NR2F2 expression increased the expression of senescence markers such as p21 and p16 in lung epithelial cells, and activated fibroblasts through epithelial-mesenchymal crosstalk, inversely overexpression of NR2F2 alleviated bleomycin-induced epithelial cell senescence and inhibited fibroblast activation. Subsequent mechanistic studies revealed that overexpression of NR2F2 alleviated DNA damage in lung epithelial cells and inhibited cell senescence. Adenovirus-mediated Nr2f2 overexpression attenuated bleomycin-induced lung fibrosis and cell senescence in mice. In summary, these data demonstrate that NR2F2 is involved in lung epithelial cell senescence, and targeting NR2F2 may be a promising therapeutic approach against lung cell senescence and fibrosis.

**Supplementary Information:**

The online version contains supplementary material available at 10.1186/s12931-024-02777-3.

## Introduction

Idiopathic pulmonary fibrosis (IPF) is an etiology unknown, chronic, progressive, destructive, and irreversible interstitial lung disease that affects over 5 million people worldwide, with a median survival of 3–5 years after diagnosis [1–3]. Various potential risk factors, including environmental exposure, genetic susceptibility, oxidative stress, viral infections, and aging-related processes, contribute to the susceptibility of this disease [4, 5]. Currently, the most widely accepted theory regarding the pathogenesis of IPF is that repeated injury to alveolar epithelial cells (AECs) by various factors leads to their abnormal activation and impaired repair. Dysregulated epithelial cells secrete multiple cytokines and growth factors, which interact with endothelial cells, mesenchymal cells, and immune cells through various signaling mechanisms, triggering the activation of fibroblasts and myofibroblasts, promoting extracellular matrix deposition, and ultimately resulting in the destruction of lung tissue architecture and decline in lung function [4]. Currently, there are only two drugs approved by the FDA for the treatment of IPF: pirfenidone and nintedanib. However, these two drugs can only moderately attenuate the decline in lung function and have certain adverse reactions [6]. Therefore, studying the pathogenesis of pulmonary fibrosis is crucial for the diagnosis and therapy of the disease.

Epidemiological investigations have revealed that IPF primarily affects individuals aged 65 and above, with the incidence rate increasing with age [7]. Furthermore, individuals aged 70 and above have a 6.9-fold higher risk of developing the disease compared to those aged 40 [8]. Therefore, IPF is considered an age-related disease.

Cellular senescence is a hallmark of aging [9, 10]. Since the pioneering observations by Hayflick and Moorhead in the 1960s, demonstrating irreversible growth arrest in normal cultured human lung fibroblasts after extensive serial passaging, an increasing body of research has revealed the widespread accumulation of senescent cells in the epithelial cells, endothelial cells, fibroblasts, and immune compartments of the human lung with advancing age. Indeed, several features of aging are intricately linked to the pathogenesis o IPF [11]. Previous studies have shown the presence of cellular senescence markers, such as increased expression of p16, p21, and elevated senescence-associated β-galactosidase activity, in fibroblasts and epithelial cells of lung tissues from IPF patients [12–14]. Additionally, lung fibroblasts derived from IPF patients exhibit an increased propensity for senescence in vitro [15, 16]. Importantly, selective removal of senescent cells has been shown to restore lung health and alleviate pulmonary fibrosis in aged mice [12]. These findings suggest that cellular senescence is not only a major driving force behind tissue and organ aging but also an independent risk factor for inducing IPF progression. In addition, according to the World Health Organization’s estimation, it is projected that by 2030, approximately one-sixth of the global population will be over 60 years [17]. Given the global population aging, the associated physical, psychological, and socio-economic burden related to IPF is substantial. However, the underlying mechanisms by which senescence potentially contributes to IPF pathogenesis remain largely elusive.

NR2F2, a member of the nuclear receptor superfamily, functions as a ligand-induced transcription factor, regulating numerous intracellular signaling pathways and participating in various physiological, pathological, and developmental processes, such as prostate cancer, breast cancer, adipogenesis, and energy metabolism [18–21]. Wu et al. found that overexpression of Nr2f2 disrupts metabolic remodeling by affecting mitochondrial function, ultimately leading to heart failure in mice [22]. In HUVEC cells, NR2F2 directly regulates the expression of E2F1 by binding to the Sp1 site in the E2F1 promoter, thereby modulating the cell cycle and influencing cell proliferation [23]. In addition, Li et al. demonstrated an increase in NR2F2 expression in mouse models of renal fibrosis induced by unilateral ischemia-reperfusion injury and unilateral ureteral obstruction, as well as in fibrotic human kidneys. Furthermore, genetic ablation of NR2F2 in mice resulted in the attenuation of injury-induced kidney fibrosis [24]. However, the functional impact of NR2F2 on cellular senescence and pulmonary fibrosis, as well as the underlying mechanisms, are still not fully understood. In this study, we aim to elucidate the role of NR2F2 in regulating cellular senescence and explore the feasibility of NR2F2 as a potential therapeutic target for IPF.

## Materials and methods

### Cell culture and treatment

The A549 cell line, MLE-12 cell line, BEAS2B cell line, and MRC-5 cell line were all purchased from American Type Culture Collection and cultured in a standard medium containing 10% fetal bovine serum, 100 U/mL penicillin, and 100 mg/L streptomycin as previously described. All cell lines were tested and confirmed to be mycoplasma-free.

### Plasmids and transfection

The human NR2F2 and mouse Nr2f2-overexpressing pcDNA3.1 plasmid or the empty pcDNA3.1 plasmid were synthesized and transfected into A549, BEAS2B and MLE-12 cells using Lipofectamine 3000 according to the manufacturer’s protocol. Human NR2F2 and mouse Nr2f2 shRNAs were designed and synthesized by Sangon Biotech and subsequently annealed and inserted into the pLKO.1 vector (Sangon, Shanghai, China).

### Western blot (WB) analysis

Western blot analysis was performed following previously described methods [25]. Briefly, lung tissues and cells were lysed in the RIPA lysis buffer (P0013B, Beyotime, Shanghai, China) supplied with protease and phosphatase inhibitors. The protein concentration of the supernatant was quantified by the BCA kit (Solarbio, Beijing, China). Equal amounts of protein were separated on 8–12% SDS-PAGE gels and then transferred onto PVDF membranes (Millipore, Darmstadt, Germany). After blocking with 5% skim milk, membranes were incubated with specific primary antibodies overnight at 4 °C. The membranes were then incubated with HRP-conjugated secondary antibody for 1 h at room temperature. Blots were visualized on the LI-COR Odyssey Fc device (LI-COR Biosciences; Lincoln, NE).

### Quantitative real-time PCR (qRT‒PCR)

For transcriptional analysis of tissue samples or cultured cells, total RNA was extracted using TRIzol (Takara, Dalian, China) as previously described [26]. cDNA was transcribed using the GoScript™ Reverse Transcription System (Promega Corporation, Wisconsin, USA). Afterward, qRT‒PCR were performed using Light Cycler 480 fluorescent quantitative PCR system (Roche) and SYBR green real-time PCR master mix (QIAGEN, Hilden, Germany) with cDNA as the template. The fold change of gene expression was calculated as 2 − ^ΔΔCt^. The primer pairs used in this study are described in Table 1.

**Table 1:** Genes selected for expression analysis

Primer name	Oligonucleotide sequence (5’-3’)
H-NR2F2-F	5’-CCTCAACTGCCACTCGTACC-3’
H-NR2F2-R	5’-CGCAAATGTTCTCGATACCC-3’
M-Nr2f2-F	5’-CGCCGAGTATAGCTGCCTCAAG-3’
M-Nr2f2-R	5’-CTGGCTCCTAACGTACTCTTCC-3’
H-CDKN1A-F	5’-GTCAGTTCCTTGTGGAGCCG-3’
H-CDKN1A-R	5’-TGGGTTCTGACGGACATCCC-3’
M-Cdkn1a-F	5’-TCGCTGTCTTGCACTCTGGTGT-3’
M-Cdkn1a-R	5’-CCAATCTGCGCTTGGAGTGATAG-3’
H-CDKN2A-F	5’-ACCAGAGGCAGTAACCATGC-3’
H-CDKN2A-R	5’-CCTGTAGGACCTTCGGTGAC-3’
M-Cdkn2a-F	5’-TGTTGAGGCTAGAGAGGATCTTG-3’
M-Cdkn2a-R	5’-CGAATCTGCACCGTAGTTGAGC-3’
H-GLB1-F	5’- CCACAGCCTGGGGTCTATAAC-3’
H-CLB1-R	5’-TGACCAACAGGTTCGCTAGAG-3’
M-Glb1-F	5’-CAAGACAGTGGCTGAAGCTCTG-3’
M-Glb1-R H-ILIB-F H-IL1B-R M-Il1b-F M-Il1b-R H-TGFB1-F H-TGFB1-R M-Tgfb1-F M-Tgfb1-R M-Fn1-F M-Fn1-R M-Col1a1-F M-Col1a1-R M-Acta2-F M-Acta2-R H-MMP12-F H-MMP12-R M-Mmp12-F M-Mmp12-R H-SERPINE1-F H-SERPINE1-R M-Serpine1-F M-Serpine1-R	5’-GAGGAAGCGTTGTTCGGTACAG-3’ 5’-CCACAGACCTTCCAGGAGAATG-3’ 5’-GTGCAGTTCAGTGATCGTACAGG-3’ 5’-TGGACCTTCCAGGATGAGGACA-3’ 5’-GTTCATCTCGGAGCCTGTAGTG-3’ 5’-TACCTGAACCCGTGTTGCTCTC-3’ 5’-GTTGCTGAGGTATCGCCAGGAA-3’ 5’-TGATACGCCTGAGTGGCTGTCT-3’ 5’-CACAAGAGCAGTGAGCGCTGAA-3’ 5’-ACAACACCGAGGTGACTGAGAC-3’ 5’-GGACACAACGATGCTTCCTGAG-3’ 5’-GATTCCCTGGACCTAAAGGTGC-3’ 5’-AGCCTCTCCATCTTTGCCAGCA-3’ 5’-CTATGCCTCTGGACGCACAACT-3’ 5’-CAGATCCAGACGCATGATGGCA-3’ 5’-GATGCTGTCACTACCGTGGGAA-3’ 5’-CAATGCCAGATGGCAAGGTTGG-3’ 5’-CACACTTCCCAGGAATCAAGCC-3’ 5’-TTTGGTGACACGACGGAACAGG-3’ 5’-CTCATCAGCCACTGGAAAGGCA-3’ 5’-GACTCGTGAAGTCAGCCTGAAAC-3’ 5’-CCTCTTCCACAAGTCTGATGGC-3’ 5’-GCAGTTCCACAACGTCATACTCG-3’

### Cell counting kit-8 (CCK8) assay

Cell viability was assessed by a CCK8 assay following the manufacturer’s instructions. In short, A549 cells were seeded at a density of 3 × 10^3^ cells/well in 96-well microplates. After indicated transfection for 48 h, 10 μL of CCK-8 solution was added to each well and incubated at 37 °C for 2 h. The absorbance was then measured at 450 nm using a microplate reader.

### EdU assay

Cell proliferation was measured by an EdU assay kit (RiboBio, Guangzhou, China). A549 cells were seeded in 96-well plates at 3 × 10^3^ cells per well. After transfection for 48 h, each well was incubated with 50 μM EdU medium for 2 h at 37 °C. The cells were then fixed in 4% paraformaldehyde for 30 min and permeabilized with 0.5% Triton X-100 for 10 min. After PBS washes, the cells were incubated with 1×Apollo reaction cocktail for 30 min. Subsequently, 1×Hoechst 33,342 was added for 30 min. The stained cells were observed and imaged using a fluorescence microscopy (Leica, Wetzlar, Germany).

### Transwell assay

The treated MRC-5 cells were digested and approximately 2 × 10^4^ cells in 100 μL serum-free DMEM were placed in the upper transwell chamber, and 600 μL medium containing 10% fetal bovine serum was added to the lower transwell chamber. The 24-well plate was incubated at 37 °C for 24 h. After 24 h incubation, the cells were fixed with 4% paraformaldehyde for 30 min, stained with crystal violet solution for 10 min, observed under electron microscope and imaged.

### Senescence-associated β-galactosidase (SA-β-gal) staining

The activity of SA-β-gal was performed by a senescence β-galactosidase staining kit following the manufacturer’s instructions (Solarbio, Beijing). Briefly, the treated cells on 24-well chamber slides and lung tissues were fixed with 4% formaldehyde for 15 min at room temperature, rinsed three times with PBS for 3 min, and then incubated with freshly prepared SA-β-Gal staining solution at 37 °C overnight. Slides were rinsed twice with PBS for 1 min at room temperature. The positive cells were observed and imaged using an electron microscope.

### Collagen gel contraction assay

Lung fibroblast contraction assay was conducted using a two-step cell contraction assay kit following the manufacturer’s guidelines (CBA-201, Cell Biolabs, Inc, San Diego, CA) as previously described [26]. The area of gel contraction was measured using ImageJ software (ImageJ 1.52q). The gel contraction rate was calculated as the contracted area divided by the initial gel release surface area.

### Mouse model of bleomycin-induced pulmonary fibrosis and IPF

The animal maintenance and handling procedures followed the Henan Normal University Institutional Animal Care and Use Committee (IACUC, SMKX-2118BS1018) guidelines, which coordinate with the Association of Animal Behavior and National Regulations. Eight-to-ten-week-old C57BL/6 N male mice were purchased from Beijing Charles River Laboratory Animal Technology Co., Ltd. (Beijing, China) and maintained in a specific pathogen-free environment. For bleomycin-induced PF, a single 50 μl injection containing 1.5 U/kg of bleomycin (Nippon Kayaku Co., Tokyo, Japan) diluted in PBS or PBS only (vehicle) was intratracheally administered. At designated time points, mice were sacrificed by intraperitoneal injection of ethyl carbamate, and samples were collected for further analysis.

IPF lung tissues and control non-IPF lung tissue samples were recruited based on the ATS/ERS/JRS/ALAT Clinical Practice Guidelines at Henan Provincial Chest Hospital. The study was approved by the Henan Provincial Chest Hospital Medical Research Ethics Committee (No. 2019-05-07), and informed consent was obtained from all the patients before surgery. The work was carried out in accordance with The Code of Ethics of the World Medical Association (Declaration of Helsinki) for experiments involving humans.

### Measurement of hydroxyproline

The lung hydroxyproline content was determined using the hydroxyproline colorimetric assay kit (MAK008, Sigma, St. Louis, MO, US) according to the manufacturer’s instructions, as previously described [26]. The results were calculated as μg hydroxyproline per right lung.

### Hematoxylin and eosin (H&E) and Masson’s trichrome staining

H&E and Masson’s trichrome staining were conducted following the protocol described in reference [26]. Mouse lung tissues were fixed in 4% paraformaldehyde for 24 h, dehydrated, and embedded in paraffin. Sections (4 μm) were routinely deparaffinized in distilled water. Morphological analysis was performed using a staining kit, following the manufacturer’s instructions, for H&E and Masson’s trichrome staining.

### Immunohistochemistry (IHC) and immunocytochemistry (ICC)

IHC was performed as previously described [1]. Briefly, the lung tissue samples were fixed with 4% paraformaldehyde, embedded in paraffin wax, and sectioned into 4 μm sections. The sections were then deparaffinized, rehydrated and treated with endogenous peroxidase blocking solution (Beyotime) for 10 min to quench endogenous peroxidase activity. Subsequently, the lung sections were accomplished with citrate buffer (Beyotime) at 100 °C for 10 min. After washing with PBS, the slides were blocked with blocking solution (Beyotime) at 37 °C for 30 min before overnight incubation with primary antibodies (anti-NR2F2). Biotin-labeled secondary antibodies (Beyotime) were applied at 37 °C for 30 min after washing. Next, the lung sections were developed with DAB working solution, counterstained with hematoxylin. Stained sections were visualized and photographed using light microscopy.

For ICC, cells were cultured on coverslips coated with poly-L-lysine, immobilized by 4% paraformaldehyde for 30 min at room temperature and permeabilized with 0.03% Triton X-100 for 5 min. After rinsing three times with PBS, the cells were blocked with 5% goat serum for 30 min, followed by incubation with primary antibodies (anti-P21, anti-γ-H2AX, anti-ki67) at 4 °C overnight and Alexa Fluor 594 (red)-conjugated secondary antibodies at 37 °C for 1 h and DAPI. The fluorescence was visualized under confocal microscope (LSM 700, Zeiss, Jena, Germany).

### Micro-CT imaging

Fourteen days after bleomycin administration, in vivo micro-CT analysis of the entire lung was conducted. Briefly, mice were lightly anesthetized with isoflurane and fixed in the supine position. Micro-CT images were acquired using a Bruker SkyScan 1276 micro-CT system (Bruker, Kontich, Belgium). The scanning parameters were set as follows: X-ray tube voltage of 60 kV and anode current of 200 μA, with a Cu filter of 0.5 mm. The total acquisition time was approximately 10 min. The reconstructed images were superimposed using Insta-Recon software (Bruker microCT, Kontich, Belgium).

### ELISA assay

The concentrations of IL-1β (Solarbio, Beijing) in mouse bronchoalveolar lavage fluid (BALF), the concentrations of TGF-β1 (Solarbio, Beijing) in the cell culture medium and the levels of 8-OHdG (Abcam, England) in cells were measured using ELISA assay kits, following the manufacturer’s instructions.

### Comet assay

Cellular DNA damage was assessed using the comet assay, following the previously described protocol [27]. In brief, cells were embedded in agarose gel and subjected to electrophoresis to allow the fragmented and denatured DNA strands to migrate, forming a comet-like pattern. Subsequently, ethidium bromide staining was performed, and the samples were observed and photographed under a fluorescence microscope for analysis (Leica, Wetzlar, Germany).

### Detection of ROS

The intracellular ROS level was assessed using a ROS assay kit (Applygen Technologies Inc., Beijing, China). Briefly, the treated A549 cells were incubated with 10 μM dihydroethidium at 37 °C for 20 min in the dark. Following incubation, the cells were washed twice with PBS carefully, and the intensity of fluorescence was determined by fluorescence microplate reader at an excitation wave length of 535 nm and emission wave length of 610 nm.

### Isolation of primary alveolar epithelial cells (AECIIs)

Primary AECIIs were isolated from the lung tissues of 2-month-old wild-type C57BL/6 mice using the protocol described in the reference [1]. Briefly, 20 mL of wash buffer was perfused into the mouse lungs, followed by the intratracheal injection of 3 mL of collagenase. The lungs were then excised, incubated at 37 °C for 1 h, and subsequently minced using a gentleMACS dissociator. The minced tissue was sequentially filtered through 100, 70, and 40 μm cell strainers, followed by gradient centrifugation (300 g, 20 min). The intermediate cells were resuspended in BEGM medium and cultured in dishes precoated with CD45/32/16. Using this protocol, AECIIs with over 80% purity were obtained and used for subsequent experiments.

### Statistical analyses

Statistical analyses were performed by GraphPad Prism 8 (GraphPad Software, Inc., San Diego, CA, USA). The Shapiro-Wilk normality test was employed to assess normal distribution. For non-normally distributed data, the Mann-Whitney U test was used to compare two groups, while the unpaired Student’s t-test was utilized for comparisons between two groups with normally distributed data. All data are shown as the mean ± standard deviation (SD) and were considered statistically significant at *P* < 0.05.

## Results

### NR2F2 was reduced in IPF and bleomycin-induced fibrotic lung epithelial cells, accompanied by increased senescent makers

**Fig. 1:**
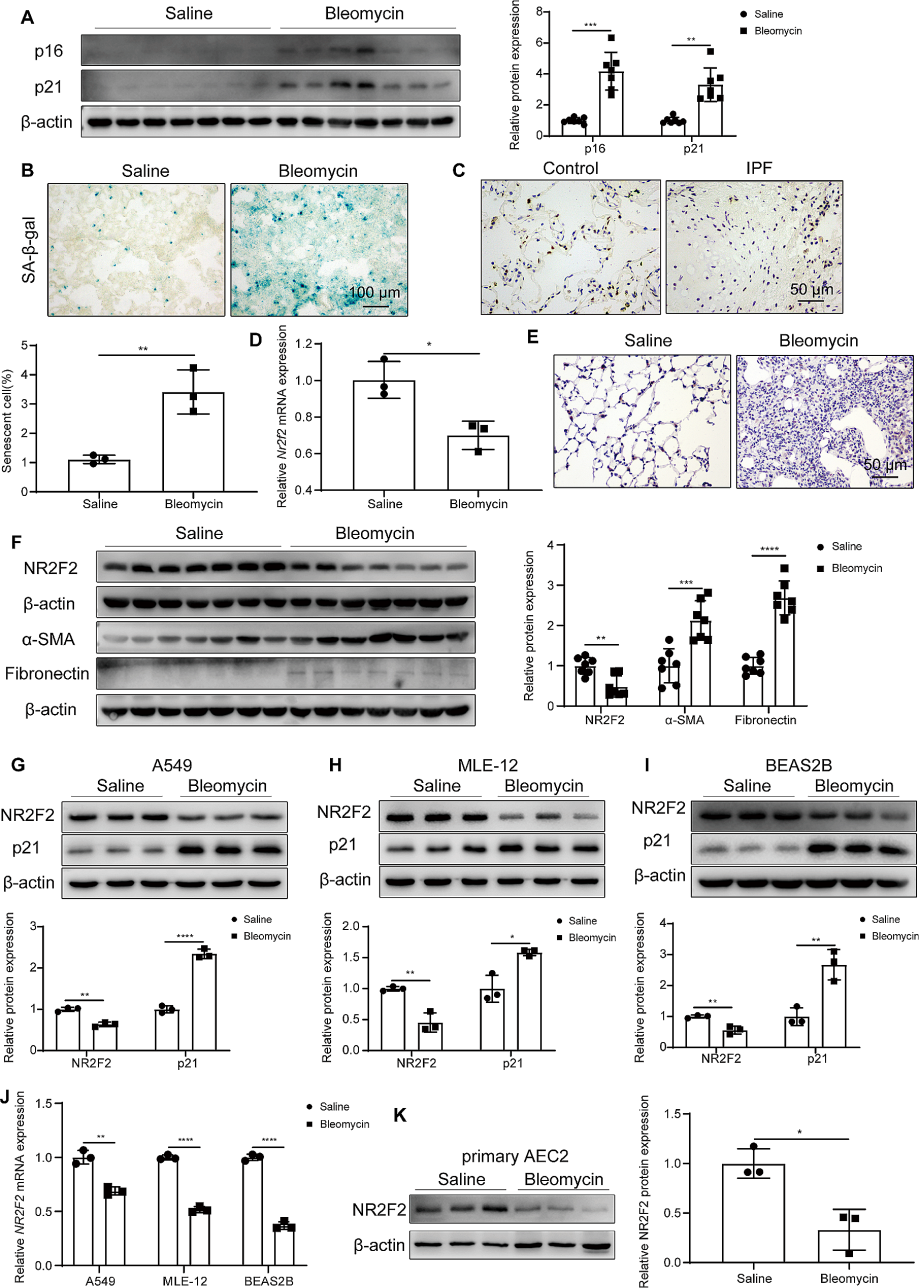
Decreased NR2F2 expression in fibrotic lungs and bleomycin-induced lung epithelial cells. (**A**) The expression levels of p21 and p16 in lung tissues from mice treated with saline or bleomycin were quantified using WB analysis. (**B**) Representative images of SA-β-Gal staining in lung tissues from mice treated with saline or bleomycin. (**C**) Representative IHC staining images of NR2F2 in lung sections obtained from control subjects and IPF patients (*n* = 3). (**D**) The transcriptional change in *Nr2f2* expression was analyzed using qRT-PCR in mice induced with saline or bleomycin. (**E**) Representative images of IHC staining using anti-NR2F2 on lung sections obtained from mice induced with saline or bleomycin. (**F**) The changes in protein levels of NR2F2, α-SMA, and Fibronectin were analyzed using WB in mice induced with saline or bleomycin. (**G**-**I**) WB analysis of NR2F2 and p21 protein expression in A549 cells (**G**), MLE-12 cells (**H**), and BEAS2B cells (**I**) treated with saline or bleomycin for 3 days. (**J**) The transcriptional changes in *NR2F2* were analyzed using qRT-PCR in A549 cells, MLE-12 cells, and BEAS2B cells induced with saline or bleomycin for 3 days. (**K**) WB analysis was performed to examine the changes in NR2F2 protein expression in primary AECs isolated from mice treated with either saline or bleomycin. Data are shown as the mean ± SD. *P<0.05, **P<0.01, ***P<0.001, ****P<0.0001

As IPF is an age-associated disease, we analyzed the expression of senescence markers and NR2F2 in fibrotic lung tissues. Firstly, we established a bleomycin-induced pulmonary fibrosis mouse model, and both hydroxyproline and micro-CT detection confirmed the successful establishment of the model (Supplementary Fig. 1B-C). Western blot results demonstrated a significant upregulation of the senescence markers p21 and p16 in the lung tissues of bleomycin-induced fibrotic mice (Fig. 1A). Concurrently, there was a notable increase in the activity of SA-β-Gal (Fig. 1B). These findings collectively indicate an enhanced cellular senescence in fibrotic lung tissues. In this context, IHC staining showed a decrease in NR2F2 protein expression in the lung tissue of IPF patient compared with the control group (Fig. 1C). In this context, IHC staining showed a decrease in NR2F2 protein expression in the lung tissue of IPF patients compared with the control group, and NR2F2 was mainly located in the alveolar epithelial cells of the control subject’s lung tissues (Fig. 1C). Meanwhile, in the lung tissues of mice treated with bleomycin compared with the control mice, both the mRNA and protein expression of NR2F2 were significantly decreased, accompanied by elevated levels of fibrosis markers, Fibronectin and α-SMA (Fig. 1D-F). Immunofluorescence results of mouse lung tissues showed downregulation of NR2F2 expression in senescent cells (Supplementary Fig. 1D). To further investigate the expression of NR2F2 in senescent cells, we utilized bleomycin to establish an epithelial cell senescence model. Given the difficulties in obtaining and sustaining primary AECs in in vitro culture, the A549 cell line is commonly used as a substitute for primary AECs. As shown in Fig. 1G, the protein expression of NR2F2 was significantly downregulated in bleomycin-treated A549 cells compared with the saline group, accompanied by a significant increase in the expression of the senescence marker p21. Similar results were also observed in MLE-12 and BEAS2B cells (Fig. 1H, I). Furthermore, in A549, MLE-12, and BEAS2B cells, bleomycin treatment significantly downregulated the mRNA levels of *NR2F2* compared with the control group (Fig. 1J). In addition, we also found a significant decrease in NR2F2 protein expression in primary AECs derived from bleomycin-treated mice compared with those derived from the saline-treated group (Fig. 1K). Together, these data indicated that NR2F2 was downregulated in fibrotic lung tissues and senescent epithelial cells, suggesting its potential involvement in cellular senescence of pulmonary fibrosis.

### NR2F2 overexpression alleviated bleomycin-induced lung epithelial cell senescence

**Fig. 2:**
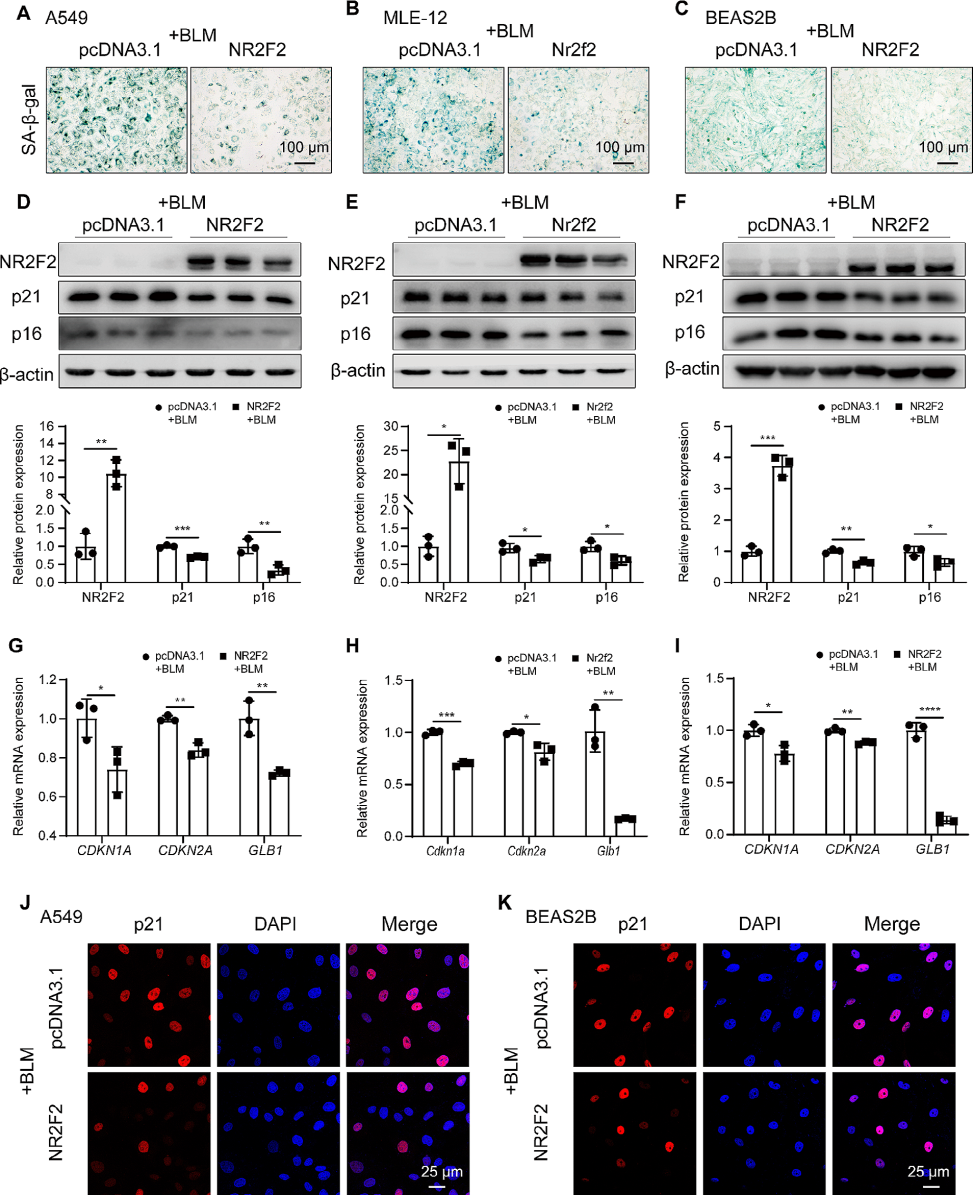
NR2F2 overexpression mitigated the senescence of lung epithelial cells induced by bleomycin. (**A**-**C**) SA-β-Gal staining was performed to detect the senescence of A549 (**A**), MLE-12 (**B**), and BEAS2B (**C**) cells treated with 0.02 U/ml bleomycin for an additional 72 h after transfection with control or NR2F2 overexpression plasmid for 48 h. (**D**-**F**) The protein expression levels of NR2F2, p21, and p16 in A549 (**D**), MLE-12 (**E**), and BEAS2B (**F**) cells treated with 0.02 U/ml bleomycin for an additional 72 h after transfection with control or NR2F2 overexpression plasmid for 48 h were measured using WB. (**G**-**I**) The mRNA expression levels of *CDKN1A* (**G**), *CDKN2A* (**H**), and *GLB1* (**I**) in A549, MLE-12, and BEAS2B cells treated with 0.02 U/ml bleomycin for an additional 72 h after transfection with control or NR2F2 overexpression plasmid for 48 h were quantified using qRT-PCR. (**J**-**K**) Representative immunofluorescence staining of p21 expression in A549 (**J**) and BEAS2B (**K**) cells transfected with control or NR2F2 overexpression plasmid for 48 h, prior to challenge with bleomycin for an additional 72 h (0.02 U/ml). Data are shown as the mean ± SD. *P<0.05, **P<0.01, ***P<0.001, ****P<0.0001

Given the above-mentioned substantial evidence indicating the downregulation of NR2F2 in bleomycin-induced mice and senescent epithelial cells, we hypothesize that upregulation of NR2F2 can alleviate cellular senescence. To validate this hypothesis, we employed an NR2F2 overexpression vector to upregulate NR2F2 expression in three immortalized lung epithelial cell lines and subsequently examined markers of cellular senescence. As expected, compared with the control group, overexpression of NR2F2 attenuated the senescence of bleomycin-induced A549, MLE-12, and BEAS2B cells, as evidenced by the elevated SA-β-Gal staining (Fig. 2A-C). Furthermore, the downregulation of senescence markers p21 and p16 at the protein level (Fig. 2D-F), as well as the decreased expression levels of *CDKN1A*, *CDKN2A*, and *GLB1* genes (Fig. 2G-I) in bleomycin-induced A549, MLE-12, and BEAS2B cells, further support this phenomenon. Since the expression of p21 protein in the nucleus is essential for cell cycle arrest, we used immunocytochemistry to investigate the impact of NR2F2 overexpression on the localization of p21 expression in bleomycin-induced epithelial cells. As shown in Fig. 2J-K, treatment with bleomycin resulted in the nuclear localization of p21 expression in A549 and BEAS2B cells. However, overexpression of NR2F2 led to a decrease in nuclear p21 expression. Additionally, overexpression of NR2F2 significantly increased the vitality and proliferative capacity of epithelial cells (Supplementary Fig. 2A-B). In conclusion, these in vitro experimental findings indicated that upregulation of the expression of NR2F2 alleviated bleomycin-induced senescence of lung epithelial cells by down-regulating expression and nuclear localization of senescence-related markers such as p21 and p16.

### NR2F2 downregulation promoted lung epithelial cell senescence

**Fig. 3:**
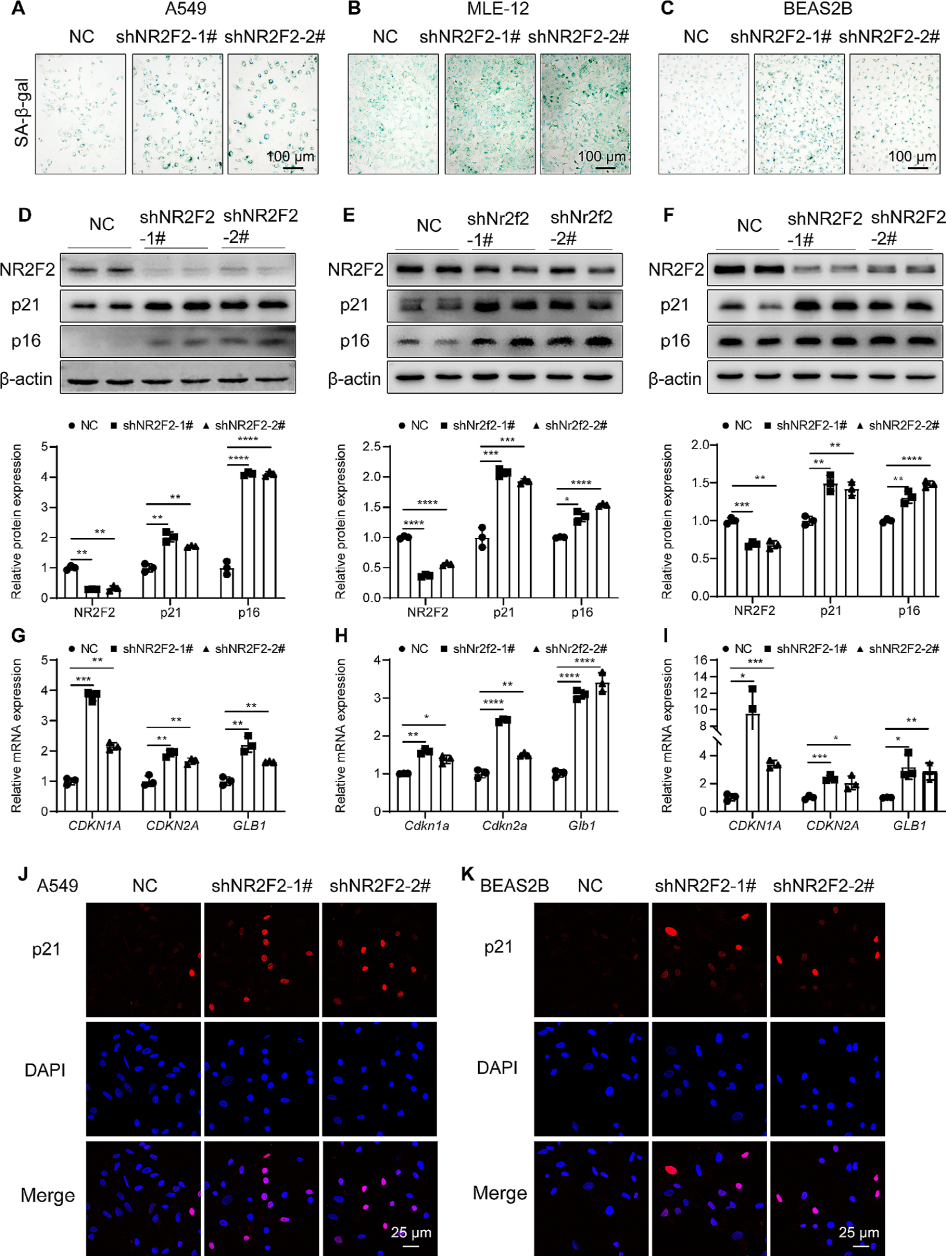
Downregulation of NR2F2 expression induced lung epithelial cell senescence. (**A**-**C**) SA-β-Gal staining was performed to assess the senescence status of A549 (**A**), MLE-12 (**B**), and BEAS2B (**C**) cells stably expressing control and NR2F2 knockdown plasmids. (**D**-**F**) The protein expression levels of NR2F2, p21, and p16 in A549 (**D**), MLE-12 (**E**), and BEAS2B (**F**) cells stably expressing control and NR2F2 knockdown plasmids were measured using WB. (**G**-**I**) The mRNA expression levels of *CDKN1A*, *CDKN2A*, and *GLB1* in A549 (**G**), MLE-12 (**H**), and BEAS2B (**I**) cells stably expressing control and NR2F2 knockdown plasmids were quantified using qRT-PCR. (**J**-**K**) Representative immunofluorescence staining of p21 expression in A549 (**J**) and BEAS2B (**K**) cells stably expressing control and NR2F2 knockdown plasmids. Data are shown as the mean ± SD. *P<0.05, **P<0.01, ***P<0.001, ****P<0.0001

To further evaluate the impact of NR2F2 expression loss on spontaneous cellular senescence, we employed shRNA to inhibit NR2F2 expression, followed by the detection of cellular senescence markers. We found that compared with the control group, downregulation of NR2F2 promoted senescence in epithelial cells, including A549 (Fig. 3A), MLE-12 (Fig. 3B), and BEAS2B (Fig. 3C), shown by elevated SA-β-Gal staining. In addition, NR2F2 knockdown significantly increased the expression of senescence markers p21 and p16 proteins (Fig. 3D-F), as well as the level of *CDKN1A*, *CDKN2*, and *GLB1* mRNA (Fig. 3G-I) in A549, MLE-12, and BEAS2B cells. To further confirm the increased senescence caused by NR2F2 depletion, we performed immunofluorescence staining on NR2F2 knockdown A549 (Fig. 3J) and BEAS-2B (Fig. 3K) cells. The results revealed a close correlation between downregulation of NR2F2 expression and increased expression of p21 in the nucleus of A549 and BEAS2B cells. Furthermore, inhibiting the expression of NR2F2 significantly reduced the viability and proliferative capacity of epithelial cells (Supplementary Fig. 2C-D). These observations indicated that the downregulation of NR2F2 lead to cellular senescence in multiple lung epithelial cell types.

### Upregulation of NR2F2 in epithelial cells inhibited lung fibroblasts activation

**Fig. 4:**
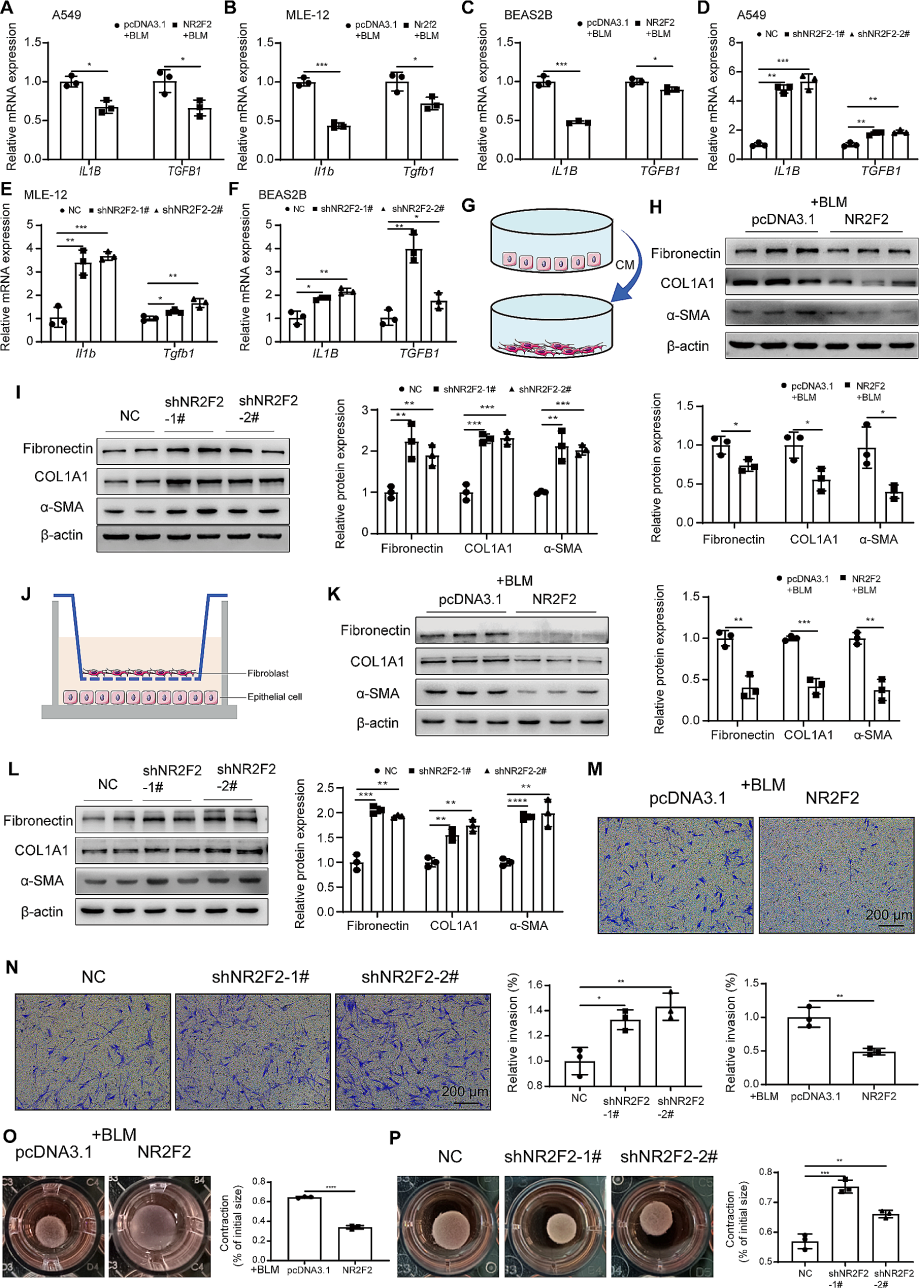
NR2F2 regulated the expression of SASP in epithelial cells and influenced fibroblast activation. (**A**-**C**) The mRNA expression levels of *IL1B* and *TGFB1* in A549 (**A**), MLE-12 (**B**), and BEAS2B (**C**) cells treated with 0.02 U/ml bleomycin for an additional 72 h after transfection with control or NR2F2 overexpression plasmid for 48 h were quantified using qRT-PCR. (**D**-**F**) The mRNA expression levels of *IL1B* and *TGFB1* in A549 (**D**), MLE-12 (**E**), and BEAS2B (**F**) cells stably expressing control or NR2F2 knockdown plasmids were quantified using qRT-PCR. (**G**) Schematic of the experimental design. The CM from A549 cells after treatment was collected and utilized for the cultivation of MRC-5 cells. (**H**) After transfection of A549 cells with either control or NR2F2 overexpression plasmids for 48 h, they were treated with 0.02 U/ml of bleomycin for an additional 72 h. Subsequently, the medium was replaced with fresh culture medium to culture for another 48 h, and the supernatant was collected and used to culture MRC-5 cells for 48 h. The protein expression levels of Fibronectin, COL1A1, and α-SMA in MRC-5 cells were measured using WB. (**I**) The culture medium of A549 cells stably expressing either control or NR2F2 knockdown plasmids was replaced with fresh medium and further incubated for 48 h. After that, the supernatant was collected and utilized to culture MRC-5 cells for 48 h. WB analysis was performed to measure the protein expression levels of Fibronectin, COL1A1, and α-SMA in MRC-5 cells. (**J**) Schematic diagram of the experimental design for the transwell co-culture system. (**K**) After transfection of A549 cells with either control or NR2F2 overexpression plasmids for 48 h, they were treated with 0.02 U/ml of bleomycin for an additional 72 h. Subsequently, MRC-5 cells were seeded in the upper transwell chamber and co-cultured with A549 cells for 48 h. The protein expression levels of Fibronectin, COL1A1, and α-SMA in MRC-5 cells were measured using WB. (**L**) MRC-5 cells were seeded in the upper transwell chamber and co-cultured with A549 cells stably expressing either control or NR2F2 knockdown plasmids in the lower transwell chamber for 48 h. WB analysis was performed to measure the protein expression levels of Fibronectin, COL1A1, and α-SMA in MRC-5 cells. (**M**, **O**) The transwell assay and collagen contraction assay were conducted to evaluate the invasive (**M**) and activation (**O**) capacities of MRC-5 cells after treatment, respectively, following the same experimental design as depicted in Fig. 4H. (**N**, **P**) The transwell assay and collagen contraction assay were performed to evaluate the invasive (**N**) and activation (**P**) capacities of MRC-5 cells after treatment, respectively (same experimental design as shown in Fig. 4I). Data are shown as the mean ± SD. *P<0.05, **P<0.01, ***P<0.001, ****P<0.0001

Senescent cells often express and secrete SASP factors, which act on neighboring cells through paracrine signaling, influencing their functional state. In this study, we found that overexpression of NR2F2 significantly reduced the mRNA levels of *IL1B*, *TGFB1* and *MMP12* induced by bleomycin in A549 (Fig. 4A, Supplementary Fig. 3A), MLE-12 (Fig. 4B, Supplementary Fig. 3B), and BEAS2B (Fig. 4C, Supplementary Fig. 3C) cells compared with the control group. However, in the absence of bleomycin induction, there was no significant difference in the expression of these genes between the control and NR2F2 overexpression groups. These results suggested that overexpression of NR2F2 can alleviate the expression of SASP induced by stress (Supplementary Fig. 3D-F). Meanwhile, overexpression of NR2F2 significantly decreased the protein levels of TGF-β1 in the culture medium of A549 and BEAS2B cells (Supplementary Fig. 4A). Conversely, the knockdown of NR2F2 expression significantly increased the mRNA levels of *IL1B* and *TGFB1* in the aforesaid cell lines (Fig. 4D-F). Correspondingly, the protein levels of TGF-β1 in the culture medium of A549 and BEAS2B cells were also upregulated (Supplementary Fig. 4B). To further assess whether senescent epithelial cells participate in the activation of lung fibroblasts through SASP, the conditioned medium (CM) from treated A549 cells was collected and used to culture MRC-5 cells (Fig. 4G). We found that compared with the control CM group, the CM collected from NR2F2 overexpressing cells significantly downregulated the expression of Fibronectin, COL1A1, and α-SMA proteins in MRC-5 cells (Fig. 4H). Conversely, the CM collected from NR2F2 knockdown cells significantly increased the expression of Fibronectin, COL1A1, and α-SMA proteins in MRC-5 cells (Fig. 4I). In order to further closely simulate the paracrine effects of cell-cell communication in vivo and create a more realistic growth environment, we employed the transwell co-culture system, which facilitates the continuous release and stimulation of paracrine mediators (Fig. 4J). Consistent with the results obtained using CM treatment, compared with the control group, the expression of fibrotic proteins Fibronectin, COL1A1, and α-SMA was significantly decreased in MRC-5 cells co-cultured with NR2F2 overexpressing A549 cells (Fig. 4K), and vice versa (Fig. 4L). Based on this, we further investigated whether this crosstalk effect between senescent epithelial cells and fibroblasts would influence the behavior of fibroblasts. As depicted in Fig. 4M and O, the CM obtained from NR2F2 overexpressing cells exhibited a significant inhibitory effect on the invasive capacity and collagen gel contraction of MRC-5 cells, compared with the control CM group. Conversely, the CM collected from NR2F2 knockdown cells significantly enhanced the invasive ability and collagen gel contraction of MRC-5 cells (Fig. 4N and P). Taken together, these data suggested that NR2F2 regulated the senescence of epithelial cells and consequently affected the activation of lung fibroblasts by influencing SASP expression and secretion.

### NR2F2 inhibited cell senescence by modulating DNA damage in epithelial cells

**Fig. 5:**
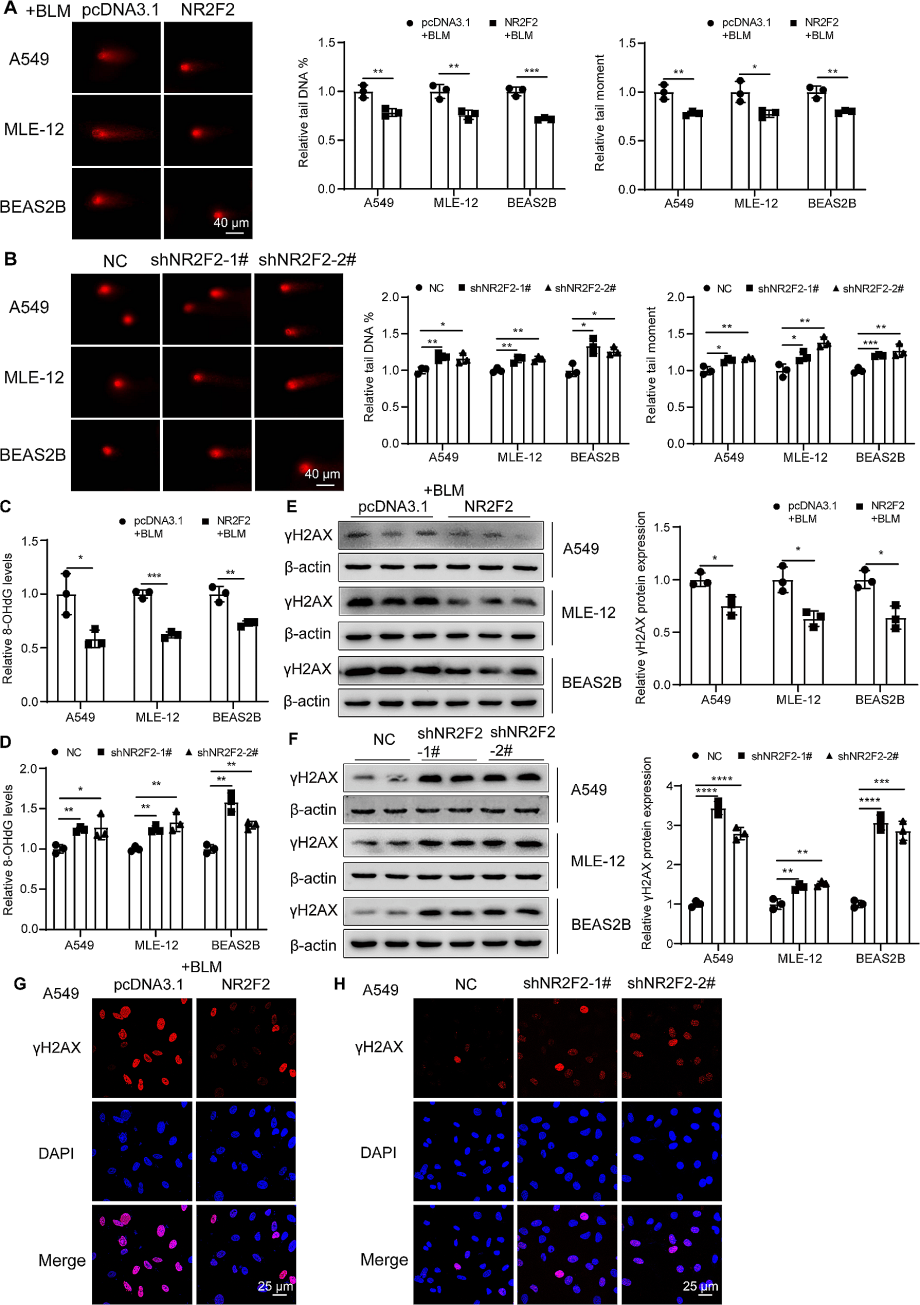
NR2F2 mitigated cell senescence by reducing DNA damage. (**A**, **C**) The comet assay (**A**) and 8-OH-dG content analysis (**C**) were used to assess the extent of DNA damage in A549, MLE-12, and BEAS2B cells after transfection with control or NR2F2 overexpression plasmid for 48 h, followed by an additional 72 h of treatment with 0.02 u/ml bleomycin. (**B**, **D**) The comet assay (**B**) and 8-OH-dG content analysis (**D**) were employed to evaluate the extent of DNA damage in A549, MLE-12, and BEAS2B cells stably expressing control and NR2F2 knockdown plasmids. (**E**) The protein expression level of γH2AX in A549, MLE-12, and BEAS2B cells treated with 0.02 U/ml bleomycin for an additional 72 h after transfection with control or NR2F2 overexpression plasmid for 48 h were quantified using WB. (**F**) The protein expression level of γH2AX in A549, MLE-12, and BEAS2B cells stably expressing control and NR2F2 knockdown plasmids were measured using WB. (**G**) Representative immunofluorescence staining of γH2AX expression in A549 cells treated with 0.02 U/ml bleomycin for an additional 72 h after transfection with control or NR2F2 overexpression plasmid for 48 h. (**H**) Representative immunofluorescence staining of γH2AX expression in A549 cells stably expressing control and NR2F2 knockdown plasmids. Data are shown as the mean ± SD. *P<0.05, **P<0.01, ***P<0.001, ****P<0.0001

Next, we further investigated the mechanism by which NR2F2 regulates cell senescence. The results of the comet assay showed that A549, MLE-12, and BEAS2B cells transfected with NR2F2 overexpression plasmid exhibited significantly lower levels of bleomycin-induced DNA damage compared with the control group. This was evident from the significant reduction in the percentage of tail DNA content and tail moment (Fig. 5A). The more severe the DNA damage, the greater the number and smaller the size of the resulting fragments, leading to an increased migration of DNA fragments towards the anode, and longer migration distances. Consequently, the length of the comet tail observed after fluorescent staining and the fluorescence intensity of DNA in the tail also increase. Conversely, knocking down NR2F2 expression significantly increased the percentage of DNA content and tail moment of A549, MLE-12, and BEAS2B cells compared with the control group (Fig. 5B). These findings suggested that NR2F2 can regulate DNA damage in lung epithelial cells. Due to the capacity of bleomycin to disrupt the molecular structure of DNA through the generation of ROS, oxidative stress can also contribute to cell senescence. In this study, we found that overexpression of NR2F2 significantly reduced the intracellular ROS levels induced by bleomycin in epithelial cells (Supplementary Fig. 5A). Conversely, interfering with NR2F2 expression increased the intracellular ROS levels (Supplementary Fig. 5B). To determine whether NR2F2 regulates cell senescence by altering ROS levels, we evaluated A549 cell senescence after ROS elimination. As shown in Supplementary Fig. 5C-D, downregulation of NR2F2 promoted A549 cell senescence, as determined by senescence-related marker p21 and p16 expression, and SA-β-gal activity. This increase was blocked by pretreatment with the ROS scavenger N-acetyl cysteine (NAC). Next, we further assessed the role of NR2F2 in oxidative DNA damage by measuring the levels of 8-OH-dG in cells. As illustrated in Fig. 5C, the overexpression of NR2F2 significantly reduced the levels of 8-OH-dG in bleomycin-induced epithelial cells. Conversely, the interference with NR2F2 expression significantly augmented the baseline levels of 8-OH-dG in A549, MLE-12, and BEAS2B cells (Fig. 5D). γH2AX, another marker of DNA damage, can label sites of double-strand breaks and recruit cell cycle checkpoints and DNA repair factors to the damaged sites. We observed a significant decrease in the protein expression of γH2AX in the aforementioned three lung epithelial cell lines with NR2F2 overexpression (Fig. 5E), and vice versa (Fig. 5F). Consistently, immunofluorescence staining demonstrated that NR2F2 overexpression significantly reduced the number of γH2AX-positive cells and γH2AX foci compared with the control (Fig. G), and vice versa (Fig. 5H). Together, all the results indicated that NR2F2 could alleviate cell DNA damage and subsequently mitigate cell senescence.

### Nr2f2 ameliorated bleomycin-induced mouse lung fibrosis

**Fig. 6:**
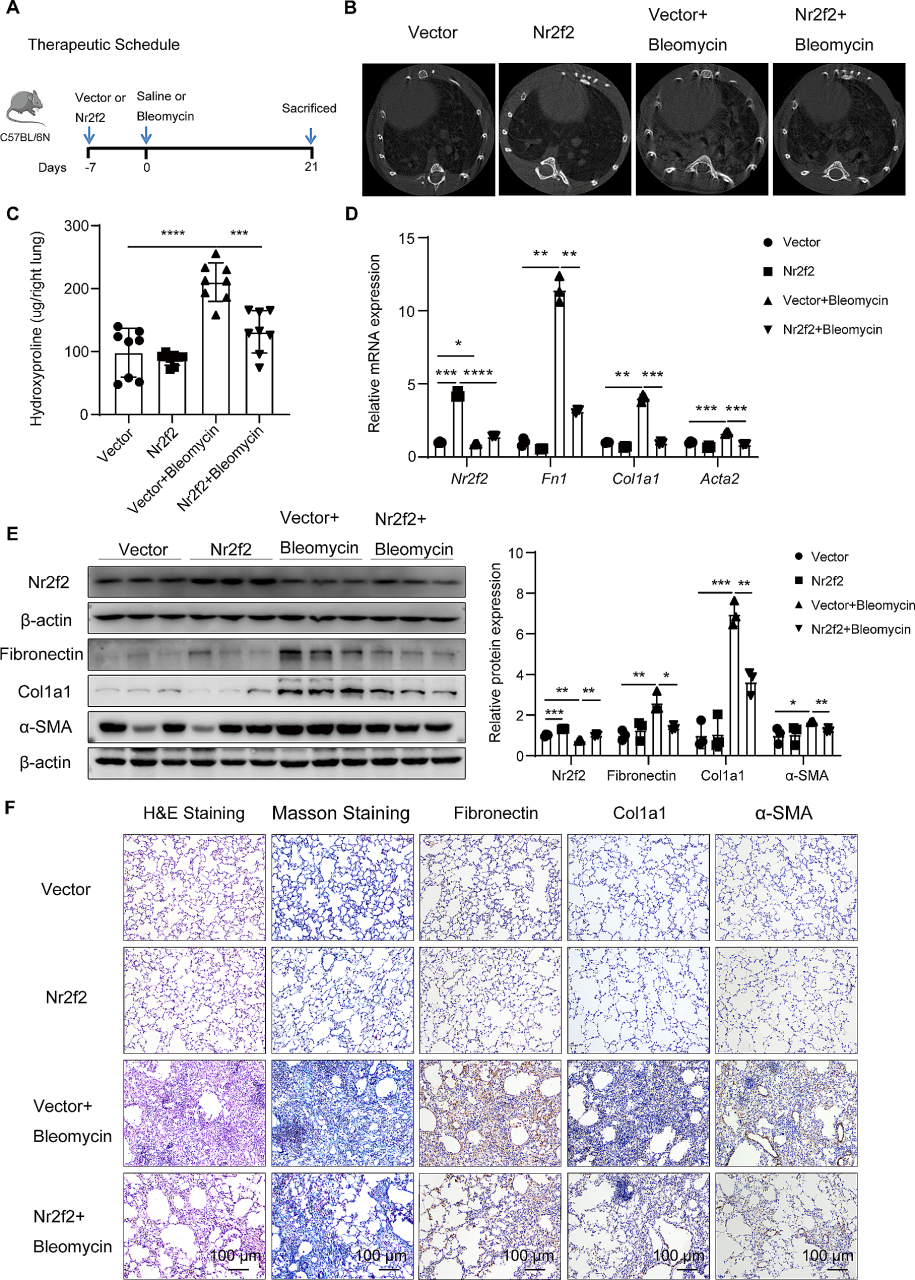
Nr2f2 overexpression attenuated bleomycin-induced mouse lung fibrosis. (**A**) The schematic diagram depicts the timeline for intratracheal instillation of AAV2/9, AAV2/9-Nr2f2, and bleomycin in a mouse model of bleomycin-induced pulmonary fibrosis. (**B**) Representative axial micro-CT images of the mouse lungs following 21 days of exposure to bleomycin. (**C**) Hydroxyproline content analysis was performed on the entire right lung of mice infected with AAV2/9 or AAV2/9-Nr2f2 treated with either saline or bleomycin. (**D**) qRT-PCR analysis the changes in gene expression of *Nr2f2*, *Fn1*, *Col1a1*, and *Acta2* in lung tissues of AAV2/9 or AAV2/9-Nr2f2 infected mice treated with either saline or bleomycin. (**E**) WB analysis the changes in protein expression of Nr2f2, Fibronectin, Col1a1, and α-SMA in lung tissues of AAV2/9 or AAV2/9-Nr2f2 infected mice treated with either saline or bleomycin. (**F**) Representative photomicrographs of H&E-staining, Masson’s trichrome-staining, and Fibronectin, Col1a1, and α-SMA IHC staining on lung sections from each group of mice. Data are shown as the mean ± SD. *P<0.05, **P<0.01, ***P<0.001, ****P<0.0001

To investigate the role of NR2F2 in the pathogenesis of pulmonary fibrosis in vivo, we induced overexpression of Nr2f2 in the lungs of C57/BL6N mice by intratracheal instillation of Adeno-associated virus serotype (AAV)2/9 or AAV2/9-Nr2f2 7 days before exposure to 1.5 U/kg bleomycin (Fig. 6A). Micro-CT imaging of mice before sacrifice revealed significant changes in lung density, characterized by dense material infiltration and increased opacity, in mice subjected to bleomycin instillation compared with the control group. However, mice overexpressing Nr2f2 exhibited improved lung tissue structure compared with mice treated with bleomycin alone, indicating reduced collagen deposition. There were no differences in lung structure between the vector and Nr2f2 overexpression groups under the saline instillation (Fig. 6B). Additionally, hydroxyproline content analysis demonstrated that Nr2f2 overexpression significantly attenuated bleomycin-induced collagen deposition compared with the vector group (Fig. 6C). Further analysis using qRT-PCR, western blot, and immunohistochemistry staining revealed that overexpression of Nr2f2 significantly reduced the expression of fibrosis markers, including Fibronectin, Col1a1, and α-SMA, in the lungs of mice following administration of bleomycin (Fig. 6D-F). Consistently, histological analysis using H&E and Masson’s trichrome staining demonstrated that overexpression of Nr2f2 significantly improved the extent of pulmonary fibrosis in mice following bleomycin instillation (Fig. 6F). Together, these results suggested that Nr2f2 overexpression alleviated bleomycin-induced pulmonary fibrosis.

### Nr2f2 attenuated bleomycin-induced cell senescence and inflammatory response in mouse lungs

**Fig. 7:**
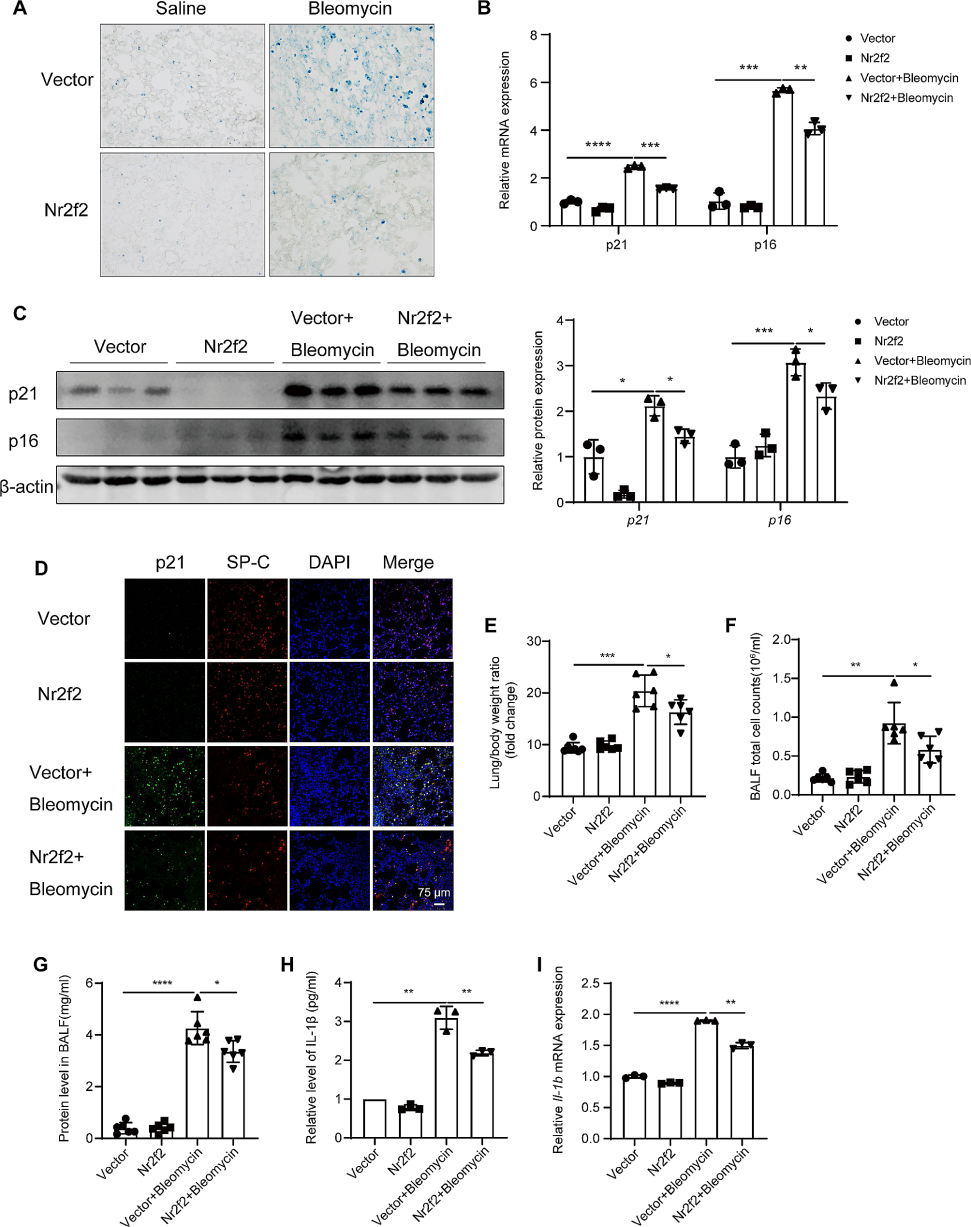
Nr2f2 overexpression ameliorated bleomycin-induced cell senescence and inflammatory response in mouse lungs. (**A**) Representative images of SA-β-Gal staining in lung tissues from mice of AAV2/9 or AAV2/9-Nr2f2 infected mice treated with either saline or bleomycin. (**B**, **C**) qRT-PCR and WB analysis the changes in gene (**B**) and protein (**C**) expression of p21 and p16 in lung tissues of AAV2/9 or AAV2/9-Nr2f2 infected mice treated with either saline or bleomycin. (**D**) Immunofluorescence analysis for SP-C (pink) and P21 (green) in lung tissues of AAV2/9 or AAV2/9-Nr2f2 infected mice treated with either saline or bleomycin. (**E**) Lung/body weight ratios were calculated in AAV2/9 or AAV2/9-Nr2f2 infected mice treated with either saline or bleomycin. (**F**-**H**) Quantification of the total number of inflammatory cells (**F**), total protein (**G**), and Il-1β content (**H**) in BALF of mice infected with AAV2/9 or AAV2/9-Nr2f2 and treated with either saline or bleomycin. (**I**) qRT-PCR analysis the mRNA expression of *Il-1β* in lung tissues of AAV2/9 or AAV2/9-Nr2f2 infected mice treated with either saline or bleomycin. Data are shown as the mean ± SD. *P<0.05, **P<0.01, ***P<0.001, ****P<0.0001

In vitro, research findings suggested that NR2F2 can regulate the senescence of lung epithelial cells and influence the activation of fibroblasts through paracrine effects. Therefore, we further evaluated whether NR2F2 has a regulatory role in cell senescence during bleomycin-induced pulmonary fibrosis in mice. As shown in Fig. 7A-C, SA-β-gal is highly expressed (Fig. 7A), accompanied by significantly elevated mRNA (Fig. 7B) and protein (Fig. 7C) expression of senescence markers p21 and p16 in the vector group of mice after bleomycin stimulation. However, these effects were rescued by overexpression of Nr2f2. This data suggested that Nr2f2 gene therapy improved bleomycin-induced cell senescence in mouse lungs. Immunofluorescence staining of mouse lung tissues showed that overexpression of Nr2f2 significantly reduced the expression of p21 in SP-C-positive cells, indicating that overexpression of Nr2f2 reduced the senescence of AT-II cells (Fig. 7D). In addition, the upregulation of Nr2f2 blunted the bleomycin-induced lung/body weight ratio (Fig. 7E). More importantly, we observed an increase in the total count of inflammatory cells in the BALF of mice following bleomycin treatment, which was consistent with the induction of total protein and Il-1β levels in the BALF. However, overexpression of *Nr2f2* resulted in a reduction in the total count of inflammatory cells in the BALF of mice, accompanied by a decrease in total protein and Il-1β levels in the BALF (Fig. 7F-H). Furthermore, the mRNA expression of *Il-1β* in mouse lung tissues showed a similar trend to the Il-1β content in the BALF (Fig. 7I). These data indicated that upregulating Nr2f2 expression not only alleviated bleomycin-induced lung cell senescence but also mitigated inflammation in the lungs.

## Discussion

IPF is a chronic, progressive, and destructive interstitial lung disease of unknown etiology, with an average age of onset exceeding 65 years and an increasing incidence with advancing age [7]. Despite the FDA approval of two drugs, pirfenidone and nintedanib, for the treatment of IPF, these medications only moderately slow disease progression and are associated with certain adverse reactions [6, 28]. Given the global aging population and cell senescence as a major driving force for tissue and organ aging, there is an urgent need to identify new targets for regulating cellular senescence [29]. In this study, we found that NR2F2 was downregulated in the lung tissue of both IPF patients and bleomycin-induced fibrotic mouse models. Silencing NR2F2 expression in lung epithelial cells increased the expression of senescence markers such as p21 and p16 and activated fibroblast activation, and vice versa. Similarly, adeno-associated virus-mediated overexpression of Nr2f2 reduced bleomycin-induced lung tissue fibrosis and senescence in mice. In addition, our previous research finding also indicated that overexpression of NR2F2 in lung fibroblasts inhibited cell activation of lung fibroblasts and extracellular matrix production [30]. Therefore, targeting NR2F2 may provide a new therapeutic strategy for combating IPF lung cell senescence and promoting fibrosis regression.

Cell senescence, recognized as a hallmark of aging, is characterized by an irreversible arrest of the cell cycle induced by a variety of intrinsic and extrinsic stimuli [31, 32]. Single-cell RNA sequencing identified multiple cell types in the lung tissue of fibrotic patients, such as epithelial cells, fibroblasts, and macrophages, displaying a senescence-like phenotype [33–36]. Yao C et al. conducted sequencing analysis of epithelial cells from both control and IPF patient fibrotic lung tissue, revealing that ATII cells isolated from the latter exhibited distinct transcriptomic features associated with cell senescence and that the senescence of ATII cells is adequate to induce lung fibrosis [34]. Additionally, Wu F et al. illustrated that the accumulation of senescence-like lung fibroblasts serves as a major pathological mechanism in lung injury caused by immune and radiation therapy [35]. Notably, the selective elimination of senescent lung fibroblasts using dasatinib plus quercetin could alleviate mouse lung fibrosis and improve lung function [12]. In addition, several studies have found that regulating the expression of certain proteins to suppress lung cell senescence could alleviate lung fibrosis. For example, the nicotinamide adenine dinucleotide hydrolase CD38 promoted alveolar epithelial cell senescence by inhibiting the activity of Sirt1 and Sirt3. Its inhibitor 78c alleviated senescence of alveolar epithelial cells, mitochondrial dysfunction, and lung fibrosis in aged mice [37]. Under various fibrotic stimuli, such as cigarette extract and bleomycin, the lack of IGFBP2 upregulated the expression of senescence markers. Conversely, intranasal delivery of recombinant IGFBP2 or overexpression of IGFBP2 reduced lung cell senescence and thereby alleviate lung fibrosis [38]. These studies collectively indicate an increase in cellular senescence in the background of lung fibrosis, and that inhibiting lung cell senescence or clearing senescent lung cells can alleviate lung fibrosis. In our study, we found that the expression of NR2F2 was significantly downregulated in the bleomycin-induced lung fibrosis mouse model and the lung epithelial cell senescence model. Inhibition of NR2F2 expression induced lung epithelial cell senescence. Conversely, NR2F2 overexpression alleviated bleomycin-induced lung epithelial cell senescence. This indicated that NR2F2 could regulate lung epithelial cell senescence and thus participate in the development of lung fibrosis.

Cell senescence, although representing a fundamentally irreversible arrest in the cell cycle, still maintains cellular viability and metabolic activity. Senescent cells can secrete various forms of SASP components, including cytokines, chemokines, bioactive lipids, extracellular matrix proteases and remodeling factors, reactive metabolites, and non-coding nucleotides, to modulate the cellular and local tissue microenvironment through both autocrine and paracrine mechanisms [17, 39]. Multiple studies have found a widespread presence of SASP in various senescent cell populations in the lungs of experimental pulmonary fibrosis models and IPF patients [40–42]. These SASP components not only serve as markers of senescence in the lung fibrosis development process but also actively participate in the senescence process and regulate the progression of pulmonary fibrosis [29]. Therefore, we further investigated whether senescent epithelial cells regulated by NR2F2 would express and secrete excessive SASP factors, thereby influencing the activation of lung fibroblasts through paracrine effects. The results showed that inhibiting the expression of NR2F2 increased the expression of *IL1B* and *TGFB1* in lung epithelial cells and promoted the secretion of TGF-β1, while conversely alleviating the expression of *IL1B* and *TGFB1* in bleomycin-induced epithelial cells and increasing the secretion of TGF-β1. Increasing evidence suggests that TGF-β1 can promote fibroblast-to-myofibroblast differentiation and excessive ECM production through activation of both canonical (Smad-based) and non-canonical (non-Smad-based) signaling pathways, thereby inducing organ fibrosis [43–45]. In this study, we found that regardless of whether lung fibroblasts were treated with CM collected from epithelial cells or co-cultured simultaneously using a transwell system, overexpression of NR2F2 in epithelial cells reduced the expression of Fibronectin, COL1A1, and α-SMA in co-cultured lung fibroblasts compared with the control group, indicating reduced activation of lung fibroblasts, and vice versa. We believe that this change in lung fibroblast activation is mediated by SASP, including TGF-β1, secreted by senescent epithelial cells. In the lungs, the extracellular matrix plays a crucial role in shaping cellular behavior in health and disease by providing cues to the cells [46]. Furthermore, changes in cytoskeletal proteins can also affect cellular function. We further evaluated the impact of changes in fibroblast activation on their behavior. The results showed that CM obtained from NR2F2-overexpressing epithelial cells significantly inhibited the invasive and collagen gel contraction abilities of lung fibroblasts compared with the control group, and vice versa. These data suggested that NR2F2 participated in the activation of lung fibroblasts and fibrosis by regulating epithelial-fibroblast crosstalk. However, the mechanism by which NR2F2 regulates epithelial cell senescence needs further elucidation.

Studies have found that during the occurrence and development of pulmonary fibrosis, various factors can induce cell senescence, such as oxidative stress, telomere attrition, oncogene activation, and ionizing radiation. In addition, DNA damage is also an inducer of cell senescence [29, 47]. The pathogenesis of pulmonary fibrosis induced by environmental factors and genetic toxic substances to some extent leads to DNA damage [48]. For example, bleomycin, a commonly used drug in rodent models of pulmonary fibrosis, can induce DNA single-strand or double-strand breaks, increase free radical production, induce oxidative stress response, leading to pulmonary inflammation and subsequent fibrosis [49]. In our study, we found that overexpression of NR2F2 reduced the production of ROS in bleomycin-induced senescent lung epithelial cells while interfering with NR2F2 expression increased ROS production in epithelial cells. Wu SP et al. reported that high expression of NR2F2 in dilated cardiomyopathy inhibited oxidative stress detoxification, leading to increased levels of ROS and exacerbating the progression of cardiac dilation [22]. Conversely, Dougherty EJ et al. demonstrated the association of NR2F2 deficiency in the vascular endothelium with cardiovascular diseases. Silencing NR2F2 expression in primary human endothelial cells resulted in increased inflammation, endothelial-mesenchymal transition, and ROS production [50]. It appears that the expression of NR2F2 is closely related to the intracellular ROS content. We speculated that NR2F2 affected cellular senescence by regulating DNA oxidative damage. Comet assays, 8-OH-dG content measurements, and expression assessments of the DNA strand break marker γH2AX conducted in three lung epithelial cell lines all showed that overexpression of NR2F2 significantly reduced bleomycin-induced DNA damage in lung epithelial cells, and vice versa. This data indicated that NR2F2 alleviated lung epithelial cell senescence and the associated fibroblast activation by reducing intracellular DNA damage. As a member of the nuclear receptor family, NR2F2 primarily exerts direct transcriptional regulation by binding to specific regulatory elements on the target gene promoter [51]. We speculate that the reduction of DNA damage caused by overexpression of NR2F2 may be attributed to the upregulation of DNA repair-related gene expression.

To transform the above in vitro findings into a more tractable therapeutic context, we determined the therapeutic effect of Nr2f2 overexpression on cell senescence in mice in vivo. The administration of AAV2/9-Nr2f2 shown that Nr2f2 overexpression alleviated bleomycin-induced lung epithelial cell senescence in mice, as determined by senescence-related marker expression, SA-β-gal activity and immunofluorescent staining. However, due to the non-cell type-specific nature of the intratracheal instillation of AAV2/9 and AAV2/9-Nr2f2 used in the in vivo animal model, it affects the entire lung. Therefore, we do not exclude the possibility that NR2F2 may improve the senescence of other cells, such as lung fibroblasts. In fact, our in vitro experimental results showed that whether co-cultured with senescent epithelial cells or directly manipulating the expression of NR2F2 in lung fibroblasts, NR2F2 can also improve the senescence of lung fibroblasts (Supplementary Fig. 6A-D). Fibrosis is closely associated with pulmonary cell senescence, and the accumulation of senescence is a key pathogenic mechanism driving the development of pulmonary fibrosis [12, 41]. Importantly, we found that overexpression of Nr2f2 also alleviated bleomycin-induced experimental pulmonary fibrosis in mice. These findings strongly suggested the potential of NR2F2 targeting as a novel therapeutic approach to cure lung fibrosis.

## Conclusion

In summary, we found that NR2F2 expression was decreased in fibrotic lung tissue and bleomycin-induced senescent lung epithelial cells. Overexpression of Nr2f2 alleviated bleomycin-induced pulmonary fibrosis. The mechanism behind this is that the high expression of NR2F2 in epithelial cells can reduce cell DNA damage, alleviate cell senescence, and thereby inhibit the activation of lung fibroblasts. This study provides strong evidence for the potential of NR2F2 as a promising target for treating pulmonary fibrosis.

### Electronic supplementary material

Below is the link to the electronic supplementary material.

Supplementary Material 1

Supplementary Material 2

## Data Availability

No datasets were generated or analysed during the current study.
