# Supplementary material for: NR2F2 alleviates pulmonary fibrosis by inhibition of epithelial cell senescence
Source: Respir Res. 2024 Apr 2;25:154. doi: 10.1186/s12931-024-02777-3 (PMC10985909; doi:10.1186/s12931-024-02777-3)

Full unedited gel for Figure 1A.

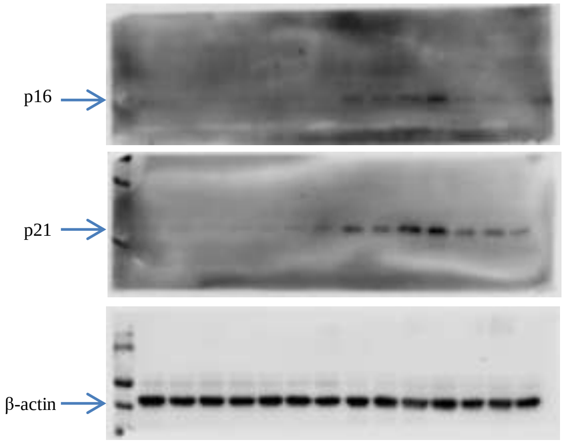

Full unedited gel for Figure 1F.

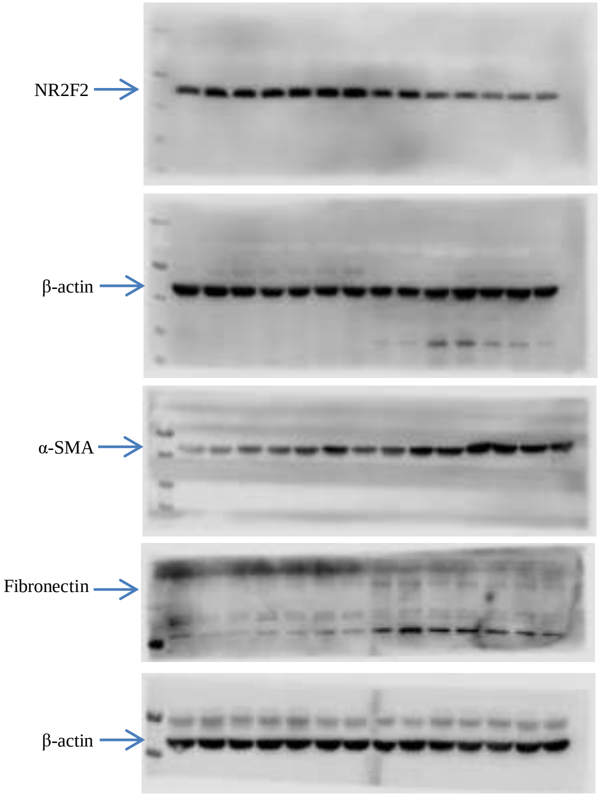

Full unedited gel for Figure 1G.

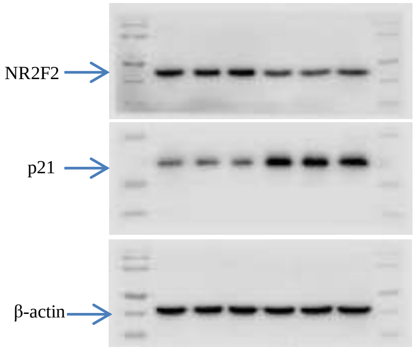

Full unedited gel for Figure 1H.

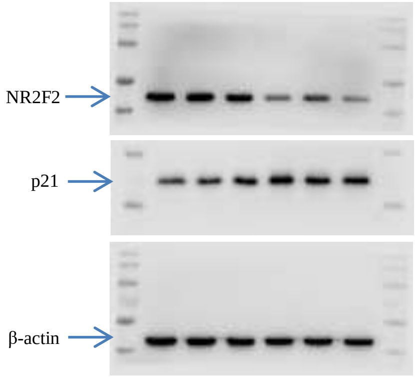

Full unedited gel for Figure 1I.

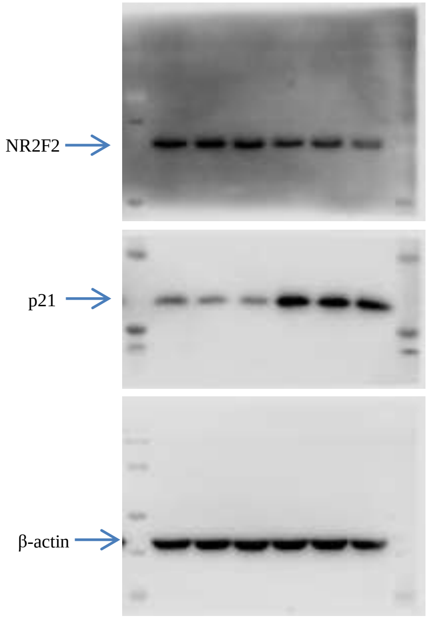

Full unedited gel for Figure 1K.

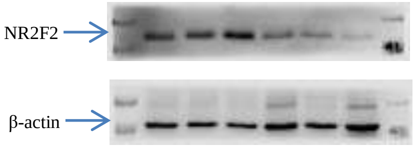

Full unedited gel for Figure 2D.

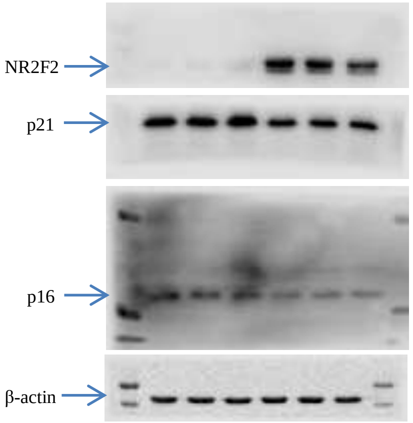

Full unedited gel for Figure 2E.

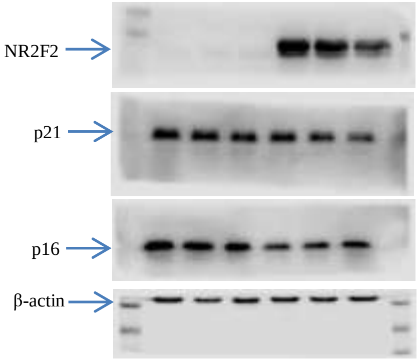

Full unedited gel for Figure 2F.

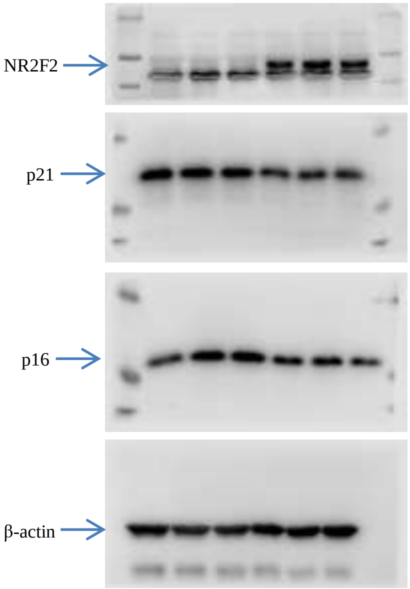

Full unedited gel for Figure 3D.

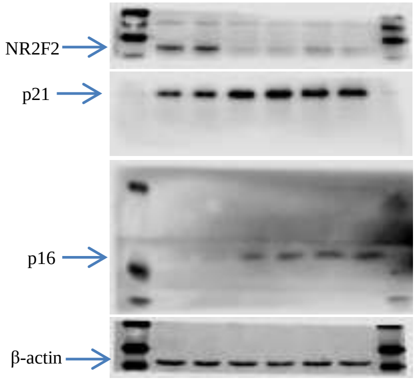

Full unedited gel for Figure 3F.

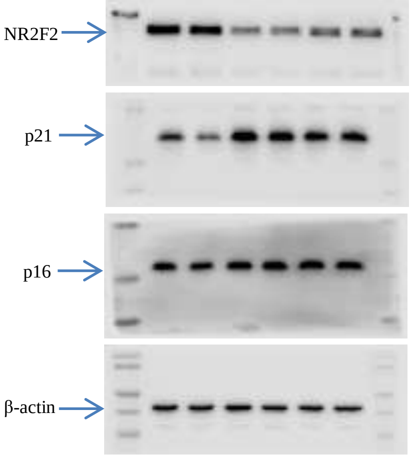

Full unedited gel for Figure 4I.

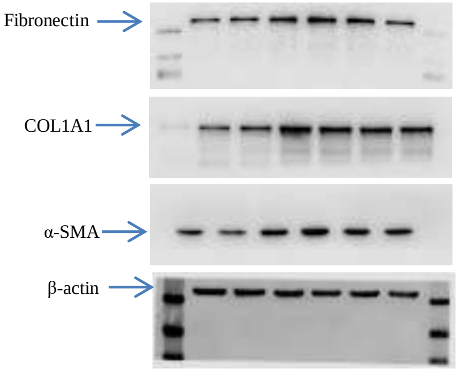

Full unedited gel for Figure 4L.

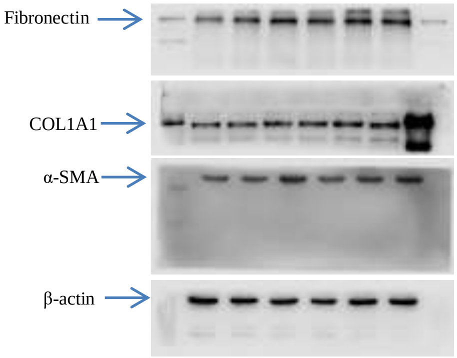

Full unedited gel for Figure 3E.

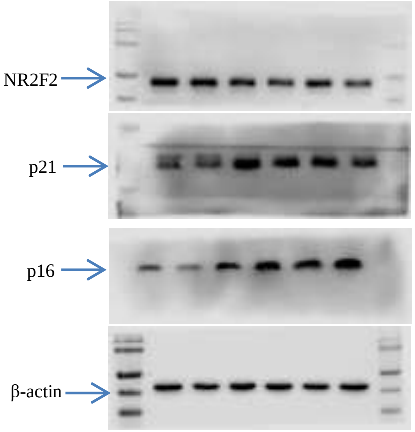

Full unedited gel for Figure 4H.

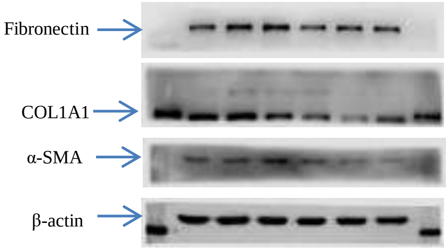

Full unedited gel for Figure 4K.

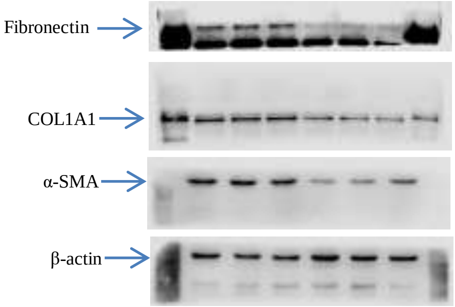

Full unedited gel for Figure 5E.

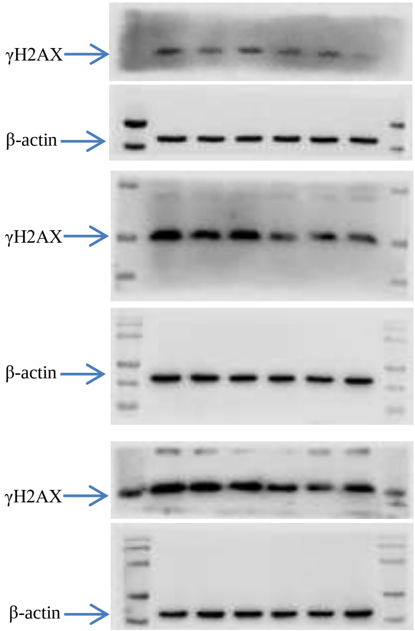

Full unedited gel for Figure 5F.

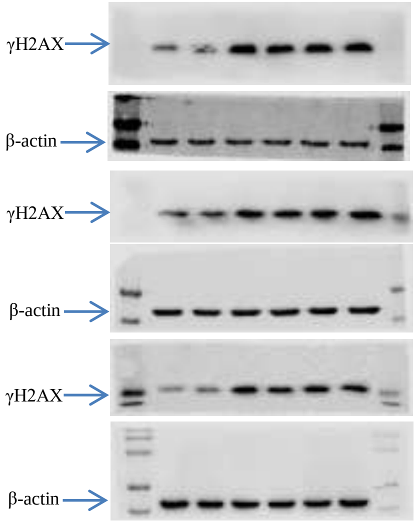

Full unedited gel for Figure 6E.

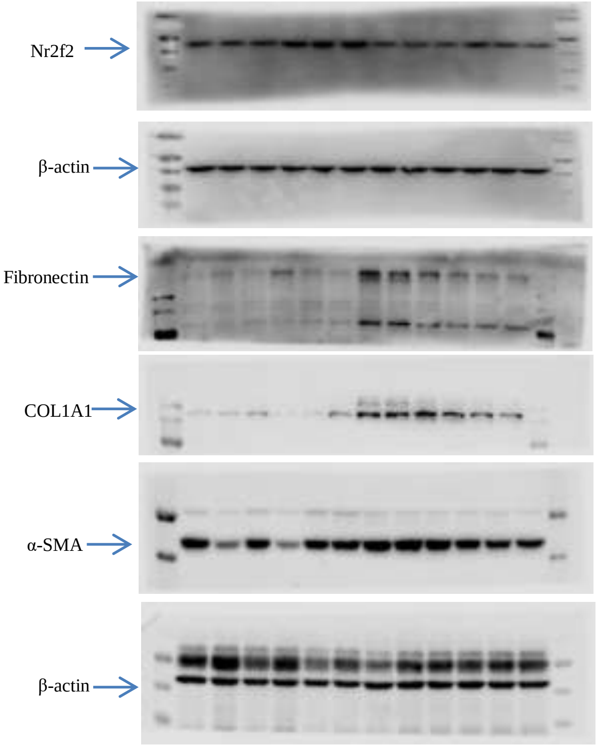

Full unedited gel for Figure 7C.

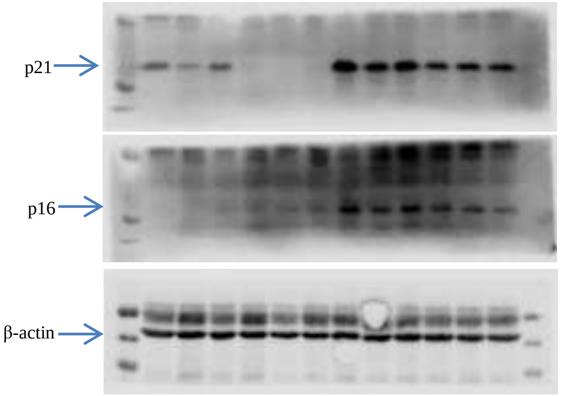

Full unedited gel for Supplementary Figure 5C.

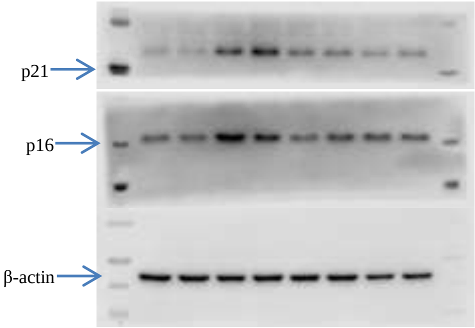

Full unedited gel for Supplementary Figure 6A.

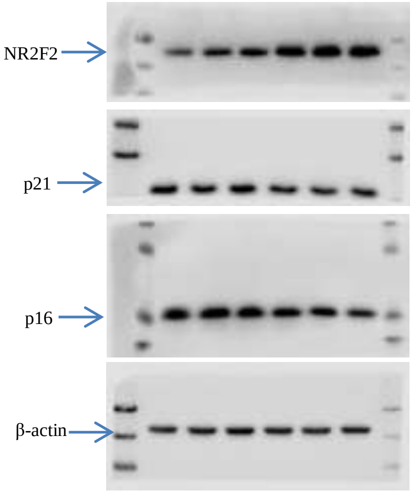

Full unedited gel for Supplementary Figure 6B.

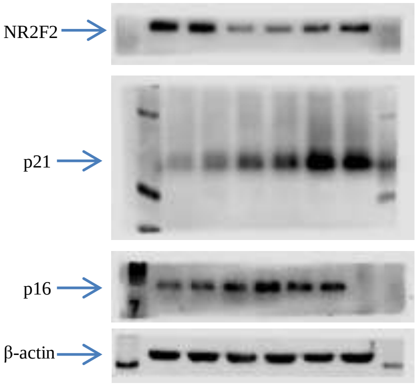

Full unedited gel for Supplementary Figure 6C.

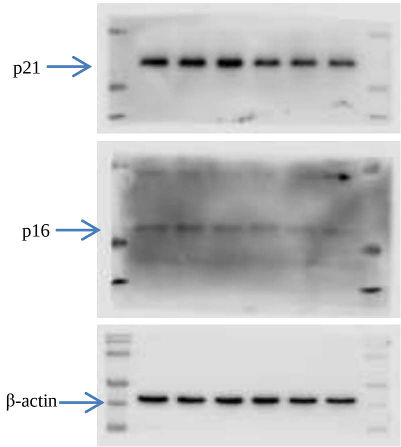

Full unedited gel for Supplementary Figure 6D.

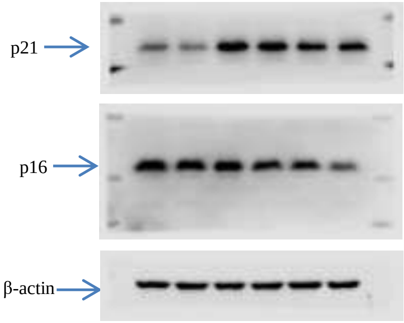

Supplement: Supplementary file 1 — Supplementary Material 1 [file 12931_2024_2777_MOESM1_ESM.pdf]
