# Supplementary material for: NR2F2 alleviates pulmonary fibrosis by inhibition of epithelial cell senescence
Source: Respir Res. 2024 Apr 2;25:154. doi: 10.1186/s12931-024-02777-3 (PMC10985909; doi:10.1186/s12931-024-02777-3)
Supplement: Supplementary file 2 — Supplementary Material 2 [file 12931_2024_2777_MOESM2_ESM.docx]

**Supplementary Figure 1**


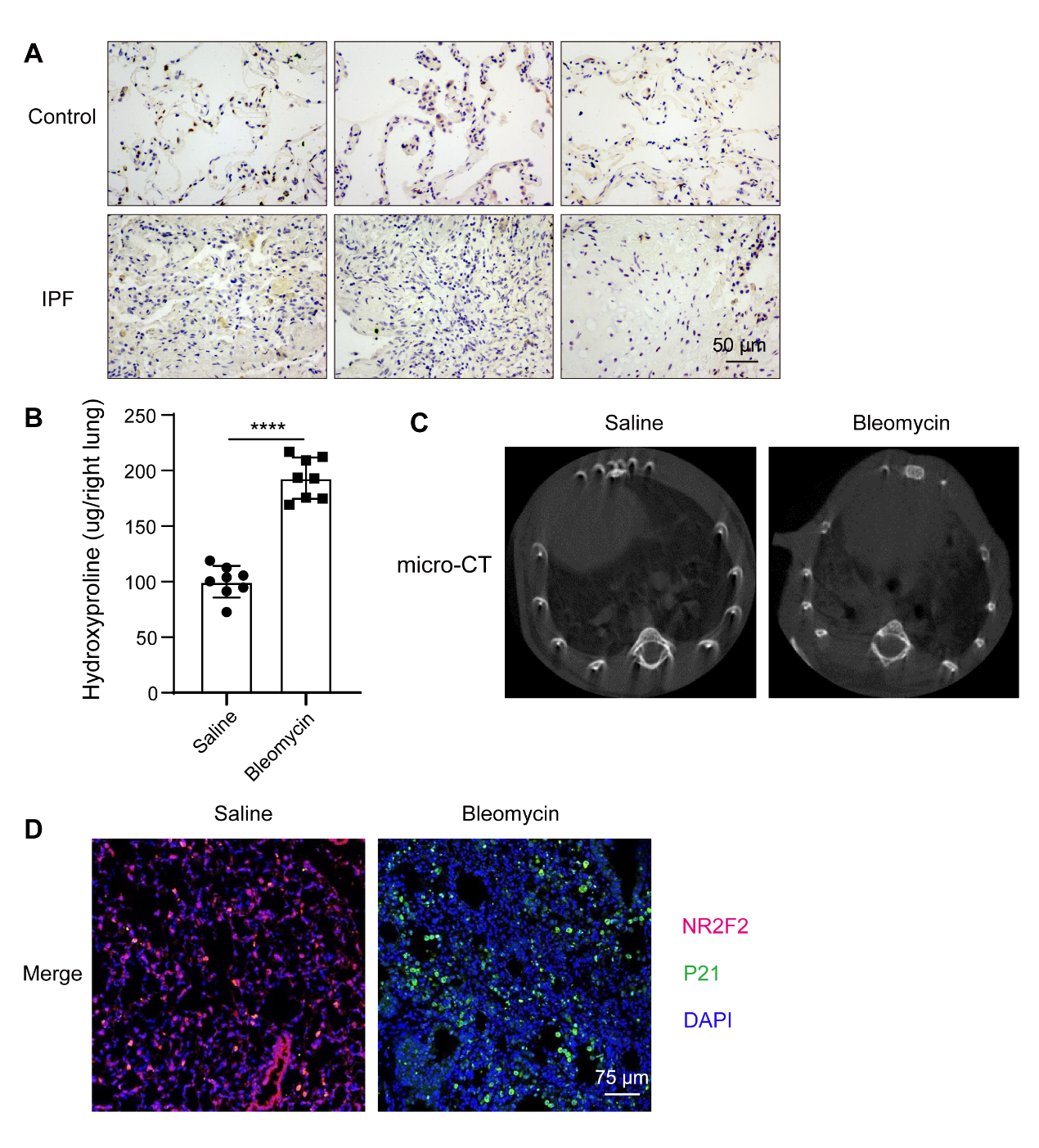


Decreased NR2F2 expression in fibrotic lungs. (A) Representative IHC staining images of NR2F2 in lung sections obtained from control subjects and IPF patients (n = 3). (B) Hydroxyproline content analysis was performed on the entire right lung of mice treated with either saline or bleomycin for 21d. (C) Representative axial micro-CT images of the mouse lungs following 21 days of exposure to bleomycin. (D) Immunofluorescence analysis for NR2F2 (pink) and P21 (green) in mice treated with saline control or bleomycin. *****P*＜0.0001.

**Supplementary Figure 2**


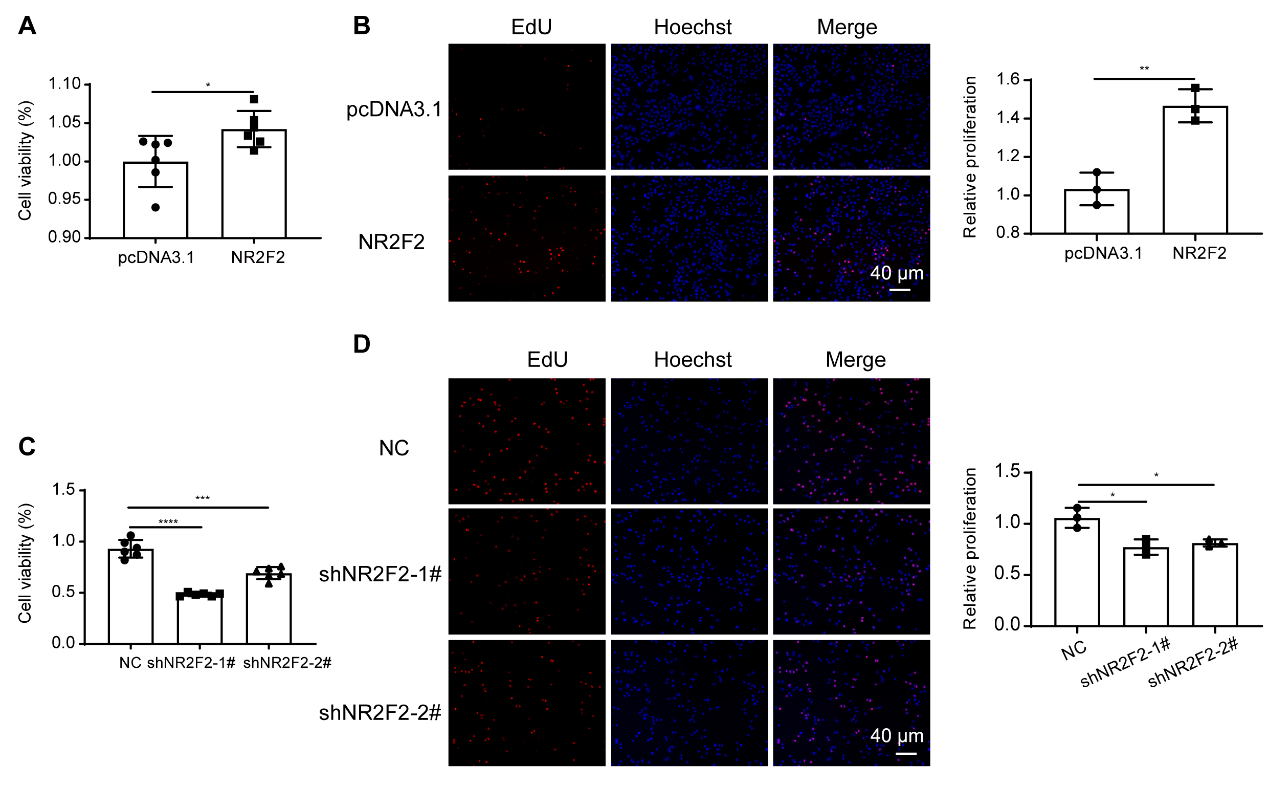
 NR2F2 affected the viability and proliferative capacity of epithelial cells. (A-B) The viability (A) and proliferative capacity (B) of A549 cells treated with 0.02 U/ml bleomycin for an additional 72 h after transfection with control or NR2F2 overexpression plasmid for 48 h using the CCK-8 and EdU assays, respectively. (C-D) The viability (C) and proliferative capacity (D) of A549 cells stably expressing control and NR2F2 knockdown plasmids using the CCK-8 and EdU assays, respectively. Data are shown as the mean± SD. **P*＜0.05; ***P*＜0.01; ****P*＜0.001; *****P*＜0.0001.

**Supplementary Figure 3**


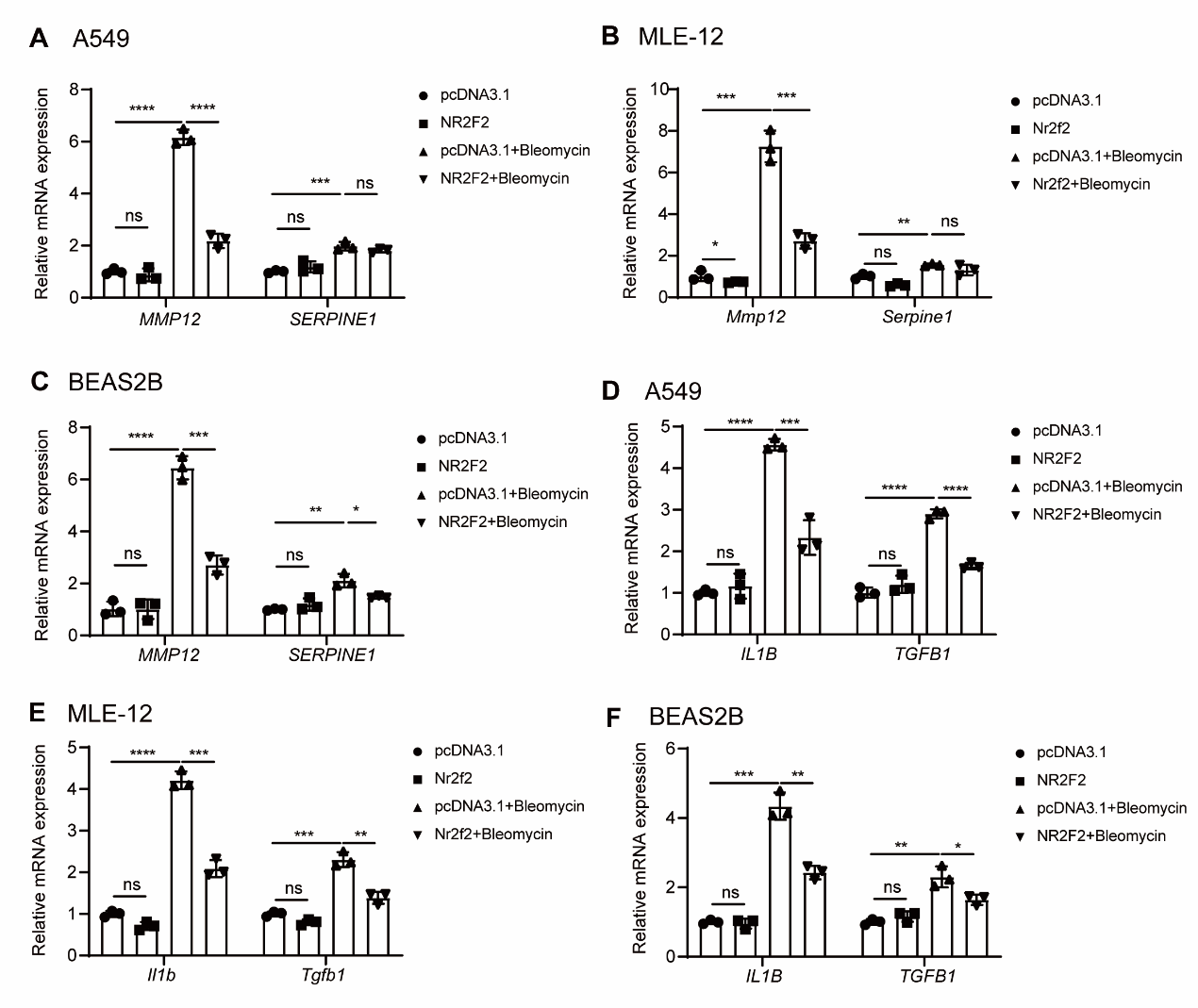


(A-C) The mRNA expression levels of *MMP12* and *SERPINE1* in A549 (A), MLE-12 (B), and BEAS2B (C) cells treated with or without 0.02 U/ml bleomycin for an additional 72 h after transfection with control or NR2F2 overexpression plasmid for 48 h were quantified using qRT-PCR. (D-F) The mRNA expression levels of *IL1B* and *TGFB1* in A549 (D), MLE-12 E), and BEAS2B (F) cells treated with or without 0.02 U/ml bleomycin for an additional 72 h after transfection with control or NR2F2 overexpression plasmid for 48 h were quantified using qRT-PCR. **P*＜0.05; ***P*＜0.01; ****P*＜0.001; *****P*＜0.0001.

**Supplementary Figure 4**


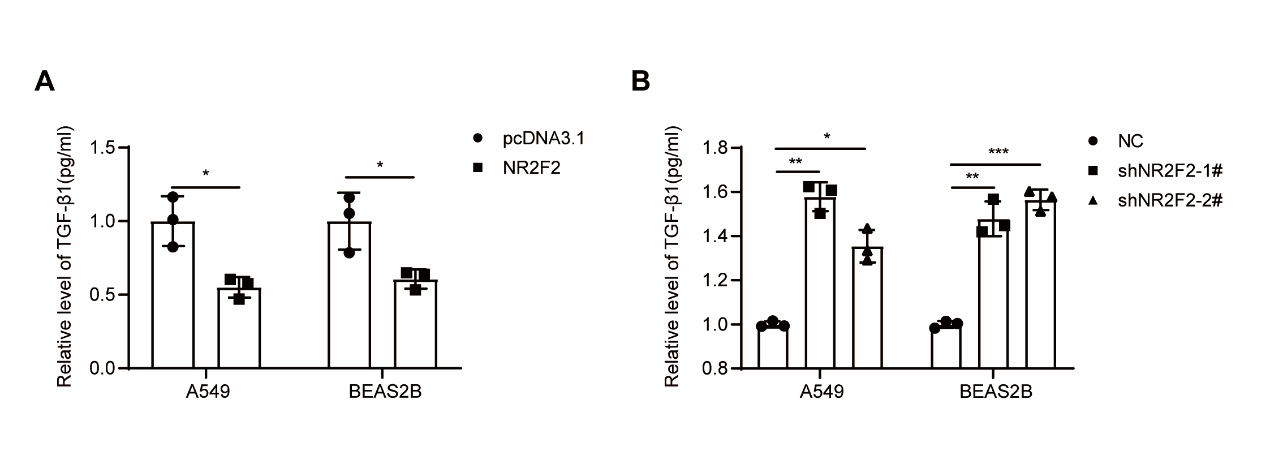


(A) After transfection of A549 and BEAS2B cells with either control or NR2F2 overexpression plasmids for 48 h, they were treated with 0.02 U/ml of bleomycin for an additional 72 h. Subsequently, the medium was replaced with fresh culture medium to culture for another 48 h, and the supernatant was collected and measured TGF-β1 content. (B) The culture medium of A549 and BEAS2B cells stably expressing either control or NR2F2 knockdown plasmids was replaced with fresh medium and further incubated for 48 h. After that, the supernatant was collected and measured TGF-β1 content. Data are shown as the mean± SD. **P*＜0.05; ***P*＜0.01; ****P*＜0.001.

**Supplementary Figure 5**


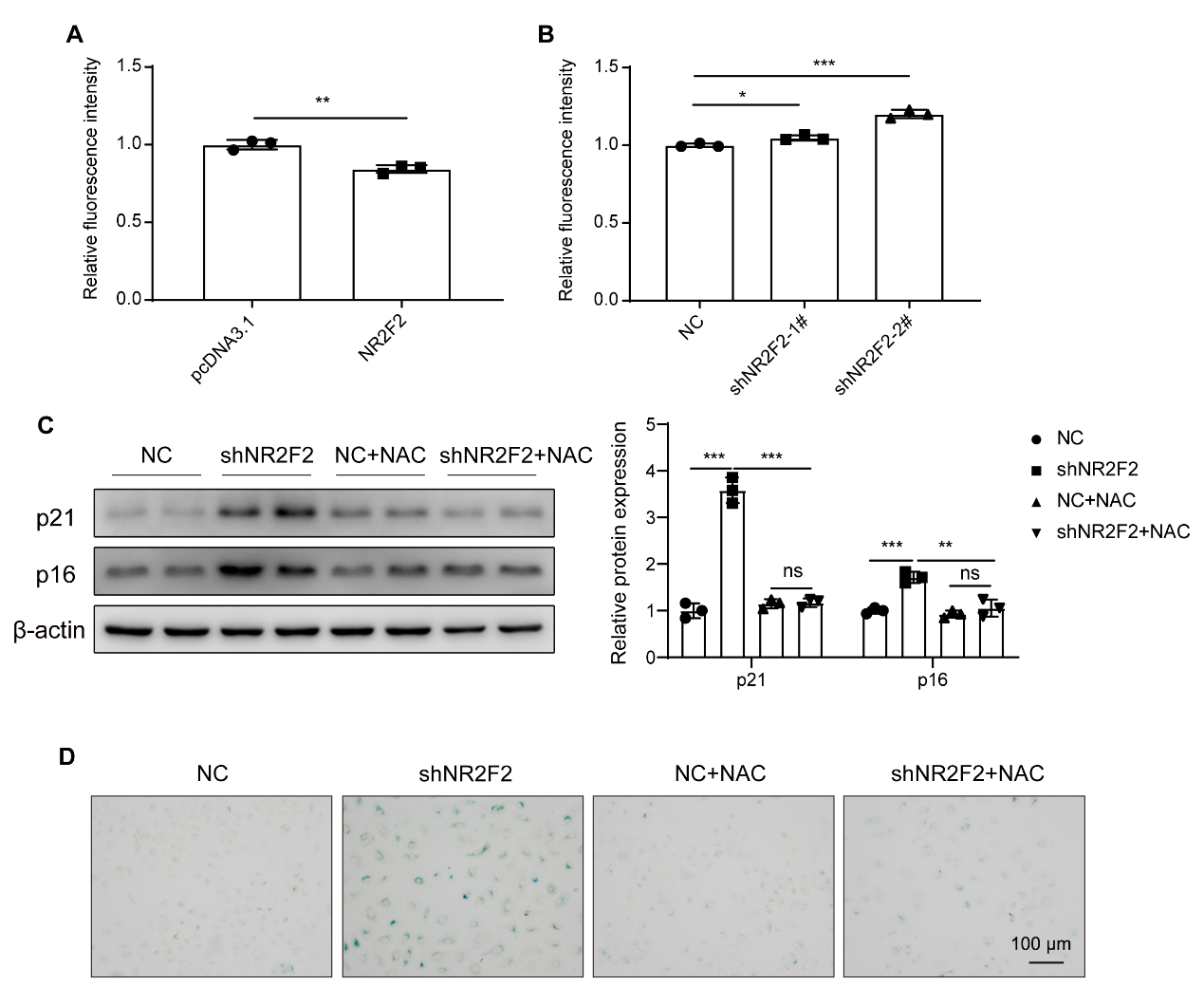


(A) The intracellular ROS level of A549 cells treated with 0.02 U/ml bleomycin for an additional 72 h after transfection with control or NR2F2 overexpression plasmid for 48 h was assessed by the dihydroethidium assay. (B) The intracellular ROS level of A549 cells stably expressing either control or NR2F2 knockdown plasmids was assessed by the dihydroethidium assay. Data are shown as the mean± SD. (C-D) Interference of NR2F2 expression in A549 cells in the presence or absence of NAC. Cell senescence was analyzed using WB (C) and SA-β-gal activity (D) analysis. **P*＜0.05; ***P*＜0.01; ****P*＜0.001.

**Supplementary Figure 6**


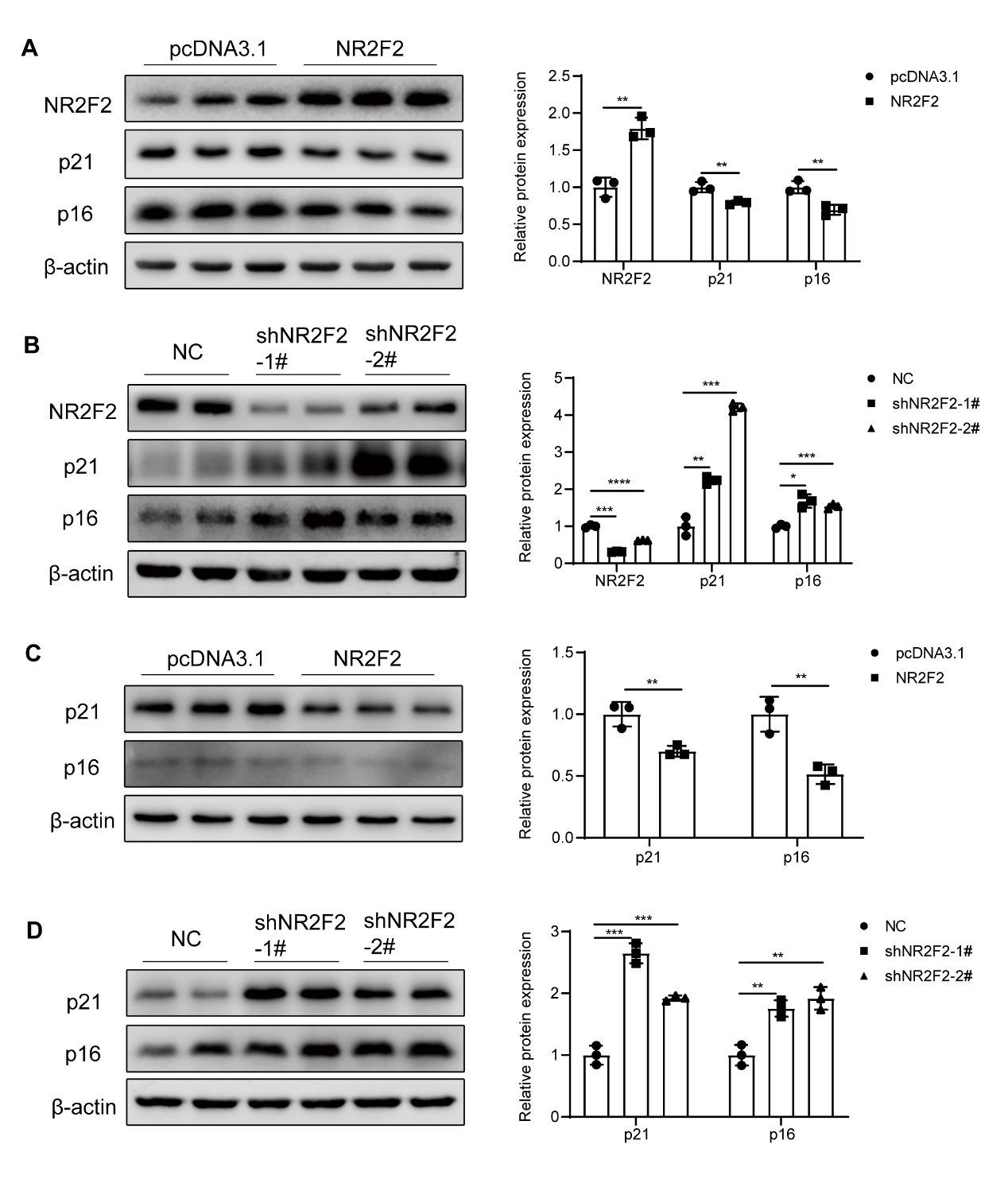


The impact of NR2F2 on lung fibroblasts. (A) The protein expression levels of NR2F2, p21, and p16 in MRC-5 cells after transfection with control or NR2F2 overexpression plasmid for 48 h were measured using WB. (B) The protein expression levels of NR2F2, p21, and p16 in MRC-5 cells stably expressing control and NR2F2 knockdown plasmids were measured using WB. (C) After transfection of A549 cells with either control or NR2F2 overexpression plasmids for 48 h, they were treated with 0.02 U/ml of bleomycin for an additional 72 h. Subsequently, the medium was replaced with fresh culture medium to culture for another 48 h, and the supernatant was collected and used to culture MRC-5 cells for 48 h. The protein expression levels of p21 and p16 in MRC-5 cells were measured using WB. (D) The culture medium of A549 cells stably expressing either control or NR2F2 knockdown plasmids was replaced with fresh medium and further incubated for 48 h. After that, the supernatant was collected and utilized to culture MRC-5 cells for 48 h. WB analysis was performed to measure the protein expression levels of p21 and p16 in MRC-5 cells.
